# Development of a subunit vaccine against the cholangiocarcinoma causing *Opisthorchis viverrini*: a computational approach

**DOI:** 10.3389/fimmu.2024.1281544

**Published:** 2024-07-10

**Authors:** Mohibullah Shah, Farva Sitara, Asifa Sarfraz, Muhammad Shehroz, Tehreem Ul Wara, Asia Perveen, Najeeb Ullah, Aqal Zaman, Umar Nishan, Sarfraz Ahmed, Riaz Ullah, Essam A. Ali, Suvash Chandra Ojha

**Affiliations:** ^1^ Department of Biochemistry, Bahauddin Zakariya University, Multan, Pakistan; ^2^ Department of Bioinformatics, Kohsar University Murree, Murree, Pakistan; ^3^ Department of Microbiology & Molecular Genetics, Bahauddin Zakariya University, Multan, Pakistan; ^4^ Department of Chemistry, Kohat University of Science & Technology, Kohat, Pakistan; ^5^ Wellman Center for Photomedicine, Harvard Medical School, Massachusetts General Hospital, Boston, MA, United States; ^6^ Department of Pharmacognosy, College of Pharmacy, King Saud University Riyadh, Riyadh, Saudi Arabia; ^7^ Department of Pharmaceutical Chemistry, College of Pharmacy, King Saud University, Riyadh, Saudi Arabia; ^8^ Department of Infectious Diseases, The Affiliated Hospital of Southwest Medical University, Luzhou, China

**Keywords:** *Opisthorchis viverrini*, cholangiocarcinoma, epitope, vaccine, parasite; immunoinformatics-based vaccine designing

## Abstract

*Opisthorchis viverrini* is the etiological agent of the disease opisthorchiasis and related cholangiocarcinoma (CCA). It infects fish-eating mammals and more than 10 million people in Southeast Asia suffered from opisthorchiasis with a high fatality rate. The only effective drug against this parasite is Praziquantel, which has significant side effects. Due to the lack of appropriate treatment options and the high death rate, there is a dire need to develop novel therapies against this pathogen. In this study, we designed a multi-epitope chimeric vaccine design against *O. viverrini* by using immunoinformatics approaches. Non-allergenic and immunogenic MHC-1, MHC-2, and B cell epitopes of three candidate proteins thioredoxin peroxidase (*Ov-TPx-1*), cathepsin F1 (*Ov-CF-1*) and calreticulin (*Ov-CALR*) of *O. viverrini*, were predicted to construct a potent multiepitope vaccine. The coverage of the HLA-alleles of these selected epitopes was determined globally. Four vaccine constructs made by different adjuvants and linkers were evaluated in the context of their physicochemical properties, antigenicity, and allergenicity. Protein-protein docking and MD simulation found that vaccines 3 was more stable and had a higher binding affinity for TLR2 and TLR4 immune receptors. *In-silico* restriction cloning of vaccine model led to the formation of plasmid constructs for expression in a suitable host. Finally, the immune simulation showed strong immunological reactions to the engineered vaccine. These findings suggest that the final vaccine construct has the potential to be validated by *in vivo* and *in vitro* experiments to confirm its efficacy against the CCA causing *O. viverrini*.

## Introduction

*Opisthorchis viverrini* (*O. viverrini*) is a trematode parasite that belongs to the family Opisthorchiidae and mostly infects fish-eating mammals (1). It is most prevalent in Southeast Asia, including Thailand, Lao PDR, Cambodia, and Vietnam (2). The life cycle of *O. viverrini* consists of two intermediate hosts, i.e., prosobranch snails (*Bithynia* spp.) as the first intermediate and cyprinid fish as the second intermediate, and one definitive host, i.e., humans (3). The parasite number is high in cyprinid fish (90–95%), whereas in snails it is less than one percent (4, 5). Humans get this infection by the ingestion of contaminated raw, undercooked, or fermented fish jammed with parasites (6). The hermaphrodite fluke, after ingestion, resides in the intrahepatic bile ducts and causes infrequent obstruction and mutilates the duct wall during the feeding process, resulting in lesions and inflammation (7, 8). Initial infection by *O. viverrini* is asymptomatic, but with the passage of time, it induces chronic inflammation and hepatobiliary complications, such as cholecystitis, cholangitis, cholangiocarcinoma, advanced periductal fibrosis, and cholelithiasis (9, 10). According to the IARC (International Agency for Research on Cancer), since 1994, *O. viverrini* is considered as a carcinogen of Group 1 because human opisthorchiasis is a risk factor for the development of cholangiocarcinoma (CCA-a bile duct cancer) (11). There are several multifactorial mechanisms by which *O. viverrini* infection results in the development of cholangiocarcinoma, but one of the most important mechanisms is the release of parasite proteins (thioredoxins, proteases, transmembrane proteins, granulin-like proteins, and calreticulin) with oncogenic potential into the hepatobiliary ducts, leading to cell proliferation and generating a tumorigenic environment (12). Carcinogenic liver fluke *O. viverrini* releases excretory secretory products (ESPs), including extracellular vehicles (EVs), that have a role in inflammation and the development of biliary cancer (13). Following infection in the bile duct, humoral, innate, and cell-mediated immune responses are invigorated by ESPs (excretory/secretory products) released by the *O. viverrini* and by evolving tissue injury. The liver fluke tackles this condition by adopting mechanisms of immune evasion and suppression. Moreover, the worm tegument can resist immunological assault and the presence of liver flukes in the hepatobiliary tracts.

Despite the severe infection caused by *O. viverrini*, there is no effective drug targeting this parasite. The only drug available against *O. viverrini* is Praziquantel (PZQ), but it is beneficial only against light infections (<1000 eggs/g of feces). Many side effects are associated with the use of Praziquantel, such as sleepiness, headache, epigastric pain, dizziness, nausea and anorexia. It is not a preventive medical procedure and produces only short-term immunity (14, 15). The widespread use of Praziquantel has led to the development of drug resistance in this trematode parasite. Moreover, it is reported that its repeated dose administration elevated the risk of CCA (16, 17). The increasing incidence of human opisthorchiasis, along with the rise in populations with PZQ-resistant parasite, vaccination presents a different approach to disease control. Currently, there is no vaccine available against infections caused by *O. viverrini* and related cholangiocarcinoma. Researchers had attempted successful vaccinations of hamster models by using recombinant forms of its different proteins and exosome-like extracellular vesicles (EVs) (18). Partial protection was noticed in hamsters when a chimeric subunit vaccine containing tetraspanin-leucine amino-peptidase was injected in hamster models against *O. viverrini* infection (19). Phumrattanaprapin et al., 2021 (20) passively immunized the hamsters by transferring monoclonal antibodies that targeted the EV protein (Tetraspanin) of *O. viverrini* resulting in hamster’s protection against parasitic infection. Chaiyadet et al., 2019 (21) vaccinated the hamster models with *O. viverrini* EVs and recombinant tetraspanins that induced the production of antibodies and reduced the parasite burden by blocking vesicle uptake by cholangiocytes. However, these vaccination studies are limited to the hamster models and are not approved yet for human trials. Therefore, there is a need of designing an effective vaccine for humans which can provide protection against *O. viverrini* infections Thioredoxins, proteases, and calreticulin proteins are considered anti-fecundity and anti-worm vaccine targets because of their involvement in the continuity of liver flukes and the development of CCA (22, 23). Developing a vaccine to prevent the pathology associated with *O. viverrini* will minimize the frequency of serious morbidities, including the emergence of CCA (18).

The advancements in the genome sequencing technologies resulted in the accessibility of various pathogenic genomes. The availability of the genomes of different pathogenic parasites allowed scientific community to apply various computational tools for determination of the candidate proteins with immunogenic potential (24, 25). The goal of this study is to design a therapeutic epitopic vaccine against *O. viverrini* by using an immunoinformatics approaches. The immunogenic CTL, HTL, and B-cell epitopes of three proteins, thioredoxin peroxidase (*Ov-TPx-1*), cathepsin F1(Ov-CF-1) and calreticulin (OvCALR), were identified and linked together through linkers to design four vaccine constructs (VCs). We also added adjuvants to the N terminal of the VCs to improve their immune response. Different analyses such as physicochemical properties, molecular docking with the immune receptors, and MD simulation, demonstrated that two constructs are worth analyzing further. These two constructs were found to elicit the human immune response and have the capability to be cloned and expressed in a suitable host cell. The vaccine constructs formulated in this study required further experimental validation to develop a proper treatment against *O. viverrini*.

## Materials and methods

**Figure 1:**
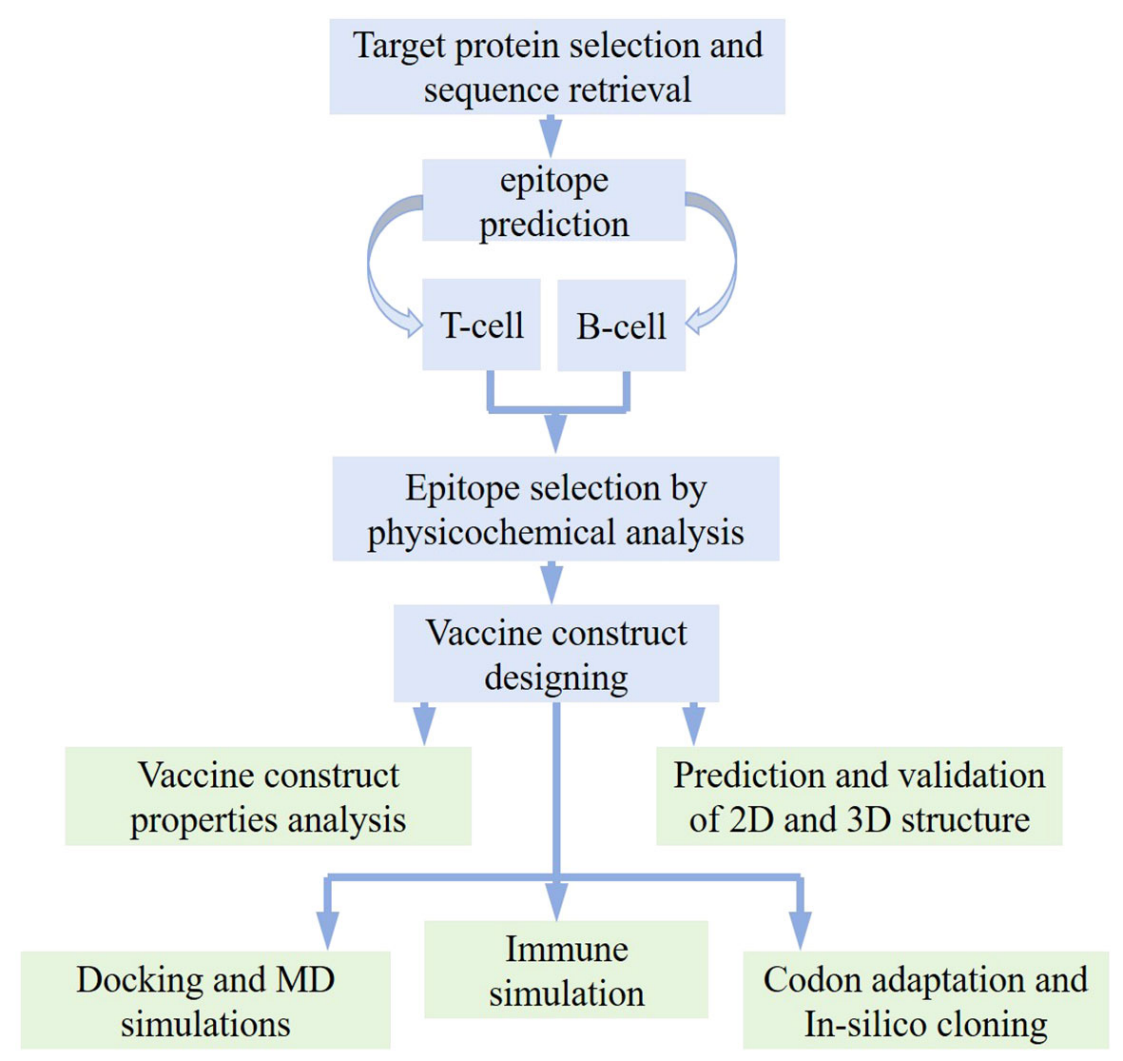
Work flow followed for designing potential vaccine against *Opisthorchis viverrini* through immunoinformatics approach.

In order to analyze the candidate proteins and design the desired vaccine, this study is pursued with updated analyses and a methodological layout as depicted in **Figure 1**.

### Protein sequence retrieval and analysis

To identify the proteins needed to design effective vaccine constructs, a comprehensive literature survey was performed. As a result, fifteen proteins of *O. viverrini* were selected, which were reported as antigenic and potential vaccine targets. Their amino acid sequences were obtained from publically available sequence databases. These proteins were assessed for their antigenic and allergenic nature using the VaxiJen server (26) and Allertop 2.0 web tools (27), respectively. Their physicochemical properties were also analyzed by the Protparam Expasy Server (28). After considering their role in carcinogenesis, high immunogenicity, high antigenicity, and non-allergenic nature, target proteins were selected for the development of several vaccine constructs.

### MHC-1 and MHC-2 epitope prediction

Non-toxic, highly antigenic, and non-allergenic epitope prediction is a crucial step in designing an effective vaccine construct. Specific site on antigenic protein molecules, called epitopes, are capable of binding with immune cell receptors to trigger an immunological response. After protein sequence retrieval and its antigenicity and allergenicity check, an epitope prediction step was carried out utilizing three distinct antigenic proteins. A free and easy-to-use database, the Immune Epitope Database (IEDB), was utilized for the prediction of T-cell epitopes (29, 30). For identification of MHC-I-restricted T lymphocytes, the IEDB-recommended NetMHCpan EL4.1 method was used, which predicts peptide binding to any MHC molecule using artificial neural networks (ANNs) (31). For MHC-I, the prediction was made over the HLA allele reference set. The predicted epitopes were ranked from lowest to highest values, and the top ten non-overlapping peptides with the lowest percentile rank were selected. The lowest percentile rank score indicates the highest binding affinity for MHC-1 cells. Furthermore, MHC-I immunogenicity was determined from IEDB with default settings.

For prediction of the MHC-II epitopes, in the NetMHC2pan 4.1 EL, a HLA reference set of 27 alleles was selected so that the epitopes for vaccine construction covered distributed worldwide HLA. The top ten non-redundant epitopes with the lowest rank value were selected. To categorize the epitopes as IFN-inducers or non-inducers, IFN-γ epitope server was used (32). This server uses cytokine models for prediction of motif, the SVM hybrid method, and IFN-γ versus. The obtained T-cell epitopes were then screened for toxicity, allergenicity, and antigenicity.

### B-cell epitope prediction

B lymphocytes are important elements of the immune system, as they promote long term immunity through the secretion of antibodies. B-cell or LBL epitopes are recognized by B cells and perform a principal role in antibody-mediated immune responses. Bepipred Linear Epitope Prediction 2.0, from IEDB, designed corresponding to the random forest technique directed on epitopes recognized from antigen-antibody structures, was utilized in the identification of linear B-cell epitopes with high antigenicity (29). The epitopes of length between 10 to 40 amino acids were selected. The selected length of epitopes assures high-affinity bonding with circulating antibodies or B-cell receptors. Predicted linear B-cell epitopes were then subjected to VaxiJen, Toxinpred, and Allertop 2.0 servers to evaluate the antigenic, toxicity, and allergenic nature of the selected epitopes.

### Vaccine construction

To develop an effective vaccine, three key fragments are required: epitopes, linkers, and adjuvants. Epitopes are peptides that are non-self, non-allergic and antigenic in nature and stimulate a particular immunological response. Linkers are short fragments of amino acids that link neighboring epitopes and adjuvants, prevent epitope folding, and enhance immune responses. To link adjuvants to B cell and T cell epitopes, different immunogenic linkers, i.e., KK, GPGPG, AAY, and EAAAK linkers, were used (33). The AAY linker is commonly used to join two proteins. This linker is essential for the structural integrity of the vaccine construct and increases the accessibility of the antigenic epitopes to immune system (34). Another commonly used, highly flexible, glycine-proline-rich linker is a GPGPG linker. It has a role in vaccine antigen proper folding and helps to eliminate the aggregation of the antigen to maintain its immunogenicity and stability. The EAAAK is an α-helical linker, often used to join adjuvants with predicted epitopes for stabilization of their linkage. Adjuvants are immunogenic substances that cause the anticipated epitopes to mount a potent immune response. To design vaccine constructs, adjuvants, HTL, CTL, and B cell epitopes were attached through different linkers at different sites. Adjuvants were joined to CTL epitopes through the EAAAK linker. AAY linkers were used to join CTL epitopes, GPGPG linkers to join HTL epitopes, and flexible KK linkers to join B cell epitopes.

### Prediction of antigenicity and allergenicity of the designed vaccine constructs

The VaxiJen v2.0 server was utilized for prediction of antigenicity of vaccine constructs using a threshold of 0.5. The accuracy of this server differs from organism to organism (26, 35). For further authentication, AntigenPro, a pathogen-dependent, sequence-based, and alignment free online tool, was used to evaluate the probable antigenicity of the vaccine constructs (36).

To predict the allergic nature of vaccine constructs, the AllerTop (https://www.ddg-pharmfac.net/AllerTOP/) server was used. It is an alignment-free technique that relies on the auto-cross-covariance transformation of amino acid sequences into a vector of uniform length and uses the chemical constituents of vaccines to identify allergenicity. The K-nearest neighbor technique is used to classify the proteins. It is based on a training set that includes 2427 known non-allergens and 2427 allergens from various species (37).

### Physicochemical properties prediction

The Expasy-ProtPram tool was used to evaluate the physicochemical properties of the vaccine which include its theoretical Pi, molecular weight, instability value, GRAVY, and aliphatic index. Molecular weight affects vaccine antigenicity; the alkaline nature of vaccine constructs is indicated by the Pi value; instability value gives vaccine stability in a test tube; thermostability is validated with the aliphatic index value (total relative volume of aliphatic side chains); polar or non-polar nature is determined by the GRAVY index; and solubility ensures equal dissolution of vaccine. The Expasy ProtPram tool works on the basis of amino acid sequences and their pKa values in the vaccine (28). The vaccine constructs with instability index score < 40 are stable. Solubility score was determined by the Protein-Sol server that uses two-stage SVM architecture to predict the tendency of proteins to be soluble upon over-expression in bacteria (38). The TMHMM v2.0 server was utilized with one line per protein output format for transmembrane helix prediction (39).

### Population coverage analysis

A particular MHC molecule binds epitopes derived from specific pathogen to form a complex that is detected by T cells (40). Different HLA alleles are distributed with varying frequencies in different ethnicities, which help in the advancement of multiple epitope vaccinology. The main goal of global population coverage is to identify the most promising vaccine epitopes for large population. The global population’s HLA allele distributions are required for multi-epitope vaccination prediction (41). Therefore, in this study IEDB population coverage server was used to analyze the population coverage of the prioritized CTL and HTL epitopes (42).

### Modelling and refinement of vaccine construct

The secondary and tertiary structural modeling of a designed constructs is an important step in vaccine design to determine its functional properties, where the 3D structures facilitate protein-protein docking analysis. SOPMA and PSIPRED webservers were used for the prediction of the 2D structures of the vaccine constructs. PSIPRED server works on two-stage position-specific iterated blast Q3 score and neural networking and provides aatypes and psipred charts of vaccine constructs that show the content of polar, non-polar, and aromatic amino acids as well as α-helix, coil, and β-strand structures (43). The SOPMA server was used for evaluation of the percentage of alpha helix, pi helix, beta turn, and random coil of the vaccine constructs.

3D modeling of the vaccine construct is an important step as it affects the propensity of the vaccine to elicit an immune response and protective immunity. The Swiss Model server (44) was used for the prediction of tertiary structure. The local and global structure quality of 3D models was improved by refining with an online, freely accessible Galaxy Refine server (45). Model validation was performed with the SAVES v6.0 server. Ramachandran plot was generated by the Vadar server (46). The plot evaluates stereochemical quality and overall structural geometry by checking the position and angles of protein residues (47). The ERRAT tool was employed for the validationof the 3D structures of vaccines. The error values are shown graphically in these results as a function of the position of a sliding 9-residue window. The error function is based on the statistics of non-bonded atom-atom interactions in the structure given. Graphic representations of error values are provided as a function of the position of a sliding 9-residue window.

### Conformational B cell epitope prediction

Protein folding can result in discontinuous B cell epitopes as it brings residues into close proximity. More than 90% of B-cell epitopes are thought to be discontinuous (48). The multi-epitope 3D structures that had been improved and confirmed were subjected to the ElliPro server to be evaluated for the existence of these epitopes. The ElliPro server employs clustering algorithms along with Thornton’s approach to execute the PI (protrusion index) value and 3D structures of each predicted epitope (48).

### Protein-protein docking and NMA evaluation

The interaction between vaccines and human immune receptors, such as TLRs, is crucial because it triggers a focused immune response against certain pathogens, generates immunological molecules that enlist and stimulate other immune cells, and may provide persistent defense against infections in the future. For this purpose, the ClusPro 2.0 server was used to determine the vaccine candidate’s interaction with TLR-2 and TLR-4 receptors on human immune cells. This server is user-friendly, reliable, and provides accurate docking results (49).

Dynamics simulation methods demonstrate the behavior of molecules in a natural environment, were employed to determine the best interaction complex with the highest docking score. The proposed vaccine design and its best-interacted receptors, the TLR2 and TLR4 complex, were examined and evaluated using the rapid and efficient iMODs server. This program predicts the range and direction of the fundamental motions of the protein-protein complex using eigenvalues, covariance, deformability, and B-factors. The server was used to perform the normal mode analysis (NMA) in internal coordinates of the atomic structures of proteins and nucleic acids (50).

### MD simulation

The Schrödinger LLC’s Desmond software was used for molecular dynamics (MD) simulation studies of the two receptors complexed with the designed vaccine (51). For every system, these simulations lasted 100 ns. Docking of the vaccine with receptors, a crucial primary step, is performed before MD simulation studies. It firstly predicts the vaccine molecules’ static binding orientation in the active sites of the both receptors. In MD simulations, the dynamic movements of individual atoms over time were simulated using Newton’s classical equations of motion (52), which allowed for the prediction of vaccine-binding interactions in a physiological setting. The vaccine-receptor complexes were prepared for simulation analysis using Schrödinger’s Maestro’s protein preparation wizard (53). Optimization, minimization, and the addition of any necessary residues that the system was lacking were all part of this preprocessing step. The solvent environment simulations employed the intermolecular interaction potential three points transferable solvent model (54). This solvent model was employed in an orthorhombic simulation box with an OPLS_2005 force field, 300 K temperature, and 1 atm pressure. In order to ensure the neutrality of the models and emulate physiological conditions, counter ions and a sodium chloride concentration of 0.15 M were introduced into the simulation systems. Before the simulations began, the models underwent a relaxation or equilibration phase in which the constraints on the system were gradually relaxed. The trajectories of the simulations were recorded and stored for subsequent analysis. Finally, root mean-square fluctuation (RMSF) and root mean square deviation (RMSD) plots were used to analyze the stability of the vaccine-receptor complexes.

### In-silico cloning and codon optimization

The Java Codon Optimization Tool (JCAT tool) was utilized for the purpose of reverse translation and codon optimization of the created vaccination sequence. It is a publically available tool that optimizes codons of a gene sequence for codon usage and gene expression in a particular host organism (55). The input was the sequence of the vaccine construct, and the *E. coli* strain K12 was used as the host for the expression as it has high reproducibility, is easy to handle, and has a larger yield volume. The options that were checked to avoid were prokaryotic ribosome binding sites, rho independent transcription terminators, and cleavage sites of restriction enzymes. JCAT purifies the vaccine sequence from invalid characters and improves its GC content and codon optimization index (CAI score). A high CAI score shows a greater expression level of vaccine in *E. coli*. Subsequently, the optimized codon sequence was inserted at the PashA1 restriction site of the pET28a (+) plasmid vector using in-silico cloning using the SnapGene restriction cloning program.

### Host immune response evaluation

For vaccine profiling and prediction of potential host immune responses, we utilized the C-ImmSim server (56). All parameters were kept at default and three injections (at time intervals 1, 84, and 168 hrs) were administered at different time periods. This tool evaluates helper T-cell1 (Th1) and helper T-cell2 (Th2) levels in addition to the generation of interferons, cytokines, and antibodies against the constructed vaccine (antigen).

### Vaccine’s safety analysis

It is essential to analyze the similarity between designed vaccine and human proteins. The reason is that any similarity among them can result in auto-immune reaction in the host (57). This similarity analysis was accomplished by pBLAST of the vaccine construct against the human proteome, which was obtained from the UniProt database.

## Results

### Sequence retrieval and physicochemical properties

Fifteen proteins, reported as antigenic and potential vaccine candidates, were selected from the literature, and their amino acid sequences were obtained via UniProtKb and NCBI databases in the FASTA format. These proteins were subjected to allergenicity and antigenicity analysis by using AllerTop v2.0 and VaxiJen v2.0 servers, respectively (**Table 1**). The theoretical Pi, aliphatic index, molecular weight, instability index, and GRAVY score were computed with the help of the Expasy ProtParam server. The TMHMM server was employed to predict the transmembrane helixes of the proteins. Of the selected 15 candidate proteins, three proteins, namely *Ov-TPx-1*, *Ov-CF-1* and *Ov-CALR*, were selected as they were highly antigenic, non-allergenic, have high stability, and a negative GRAVY value (**Table 1**). These three vaccine candidate proteins were subjected to different analysis and the development of vaccine constructs.

**Table 1:** Physicochemical properties analysis of selected antigenic proteins.

Sr No.	IDs	Proteins	No. of A. A	Mol. weight	Theoretical PI	Instability index	Aliphatic index	GRAVY	Antigenicity	Allergenicity	Topology value
**1.**	**A0A1P8P1U1**	**Calreticulin**	**415**	**48044.8**	**4.56**	**32.28 (stable)**	**58.51**	**-1.061**	**0.6564 (antigen)**	**Non-allergen**	**0**
2.	A0A075A0M6	fructose‐1,6‐bisphosphate aldolase	576	63860.27	6.15	39.91 (stable)	97.36	-0.122	0.3118 (non-antigen)	Non-allergen	0
3.	A0A074Z863	Triosephosphate isomerase	252	27790.9	6.4	41.21 (unstable)	84.76	-0.243	0.5630 (antigen)	Allergen	0
4.	B8XSI4	Granulin‐like proteins	102	10971.92	8.29	45.85 (unstable)	83.24	0.233	0.7807 (antigen)	Non-allergen	0
5.	A0A074ZCD5	Cathepsin B1	337	37610.46	5.28	46.81 (unstable)	61.96	-0.428	0.3836 (non-antigen)	Non-allergen	0
**6.**	**A0A074YV15**	**Cathepsin F1**	**232**	**26605.4**	**4.45**	**36.85 (stable)**	**57.59**	**-0.788**	**0.5627 (antigen)**	**Non-allergen**	**0**
7.	A0A074ZCI7	Tetraspanin protein-1	252	28668.37	5.11	37.61 (stable)	101.71	0.343	0.6300 (antigen)	Non-allergen	3
8.	A0A074Z349	Tetraspanin protein-2	223	23884.56	5.03	37.03 (stable)	129.69	0.935	0.5422 (antigen)	Non-allergen	4
9.	A0A075A5E5	Thioredoxin	273	31720.28	8.93	55.69 (unstable)	97.84	0.197	0.4145 (non-antigen)	Non-allergen	2
**10.**	**B4Y9T5**	**Thioredoxin peroxidase**	**212**	**23570.3**	**6.59**	**36.92 (stable)**	**88.77**	**-0.053**	**0.5258 (antigen)**	**Non-allergen**	**0**
11.	H2KVT7	Glutathione S transferase	213	24552.38	5.22	38.22 (stable)	76.9	-0.524	0.4629 (non-antigen)	Non-allergen	0
12.	A0A4V3SEV8	Thioredoxin glutathione reductase	631	69709.03	8.3	31.22 (stable)	84.53	-0.272	0.4385 (non-antigen)	Non-allergen	0
13.	A0A074Z7C4	Glutathione	342	37747.49	6.91	56.43 (unstable)	67.54	-0.592	0.6565 (antigen)	Non-allergen	0
14.	A0A074YWU5	Thioredoxin & glutathione reductase	432	47767.22	5.54	25.03 (stable)	82.18	-0.261	0.3762 (non-antigen)	Non-allergen	0
15.	A0A075A5Q8	Glutaredoxin	921	105572.8	6.47	40.93 (unstable)	93.12	-0.14	0.4609 (non-antigen)	Non-allergen	0

The rows in bold show the selected vaccine proteins.

### T-cell epitopes evaluation

Cytotoxic T lymphocytes (CTL) and helper T lymphocytes (HTL) play roles in generating a long-lasting immune response against pathogens by inactivating exotic or non-self-molecules and producing specific immunoglobulins or antibodies, respectively. The IEDB-recommended NetMHCpan EL 4.1 method was used for highly immunogenic CTL epitope prediction. From the generated epitopes, the top ten non-overlapping epitopes having a low percentile rank score were sorted out for each selected protein. The prioritized epitopes were then subjected to antigenicity, allergenicity, and immunogenicity analyses. To formulate the vaccines, a total of four CTL epitopes from three proteins were picked based on their non-allergenicity, high antigenicity, and high immunogenicity scores. The epitopes were LFYPLDFTF and EEGHAFRGQF from *Ov-TPx-1*, ALYEEFKLK from *Ov-CF-1*, and KTSADARYY from *Ov-CALR* (**Supplementary Tables 1**–**3**).

For HTL epitope prediction, the sequences of the three proteins were submitted to the IEDB-recommended NetMHC2pan 4.1 EL method. We selected nine, eight, and ten epitopes from *Ov-TPx-1, Ov-CF-1*, and *Ov-CALR* proteins, respectively, from the predicted dataset of epitopes. These epitopes were shortlisted according to low percentile scores and non-overlapping peptide sequence criteria. The allergenicity, antigenicity, and IFN-inducing characteristics of these epitopes were evaluated by using Allertop 2.0, VaxiJen 2.0, IFN-epitope, IL4pred, and IL10 servers, respectively. Finally, on the basis of non-allergenicity and a high antigenic score, we selected three HTL epitopes of *Ov-TPx-1* (KNISLKDYRGKYVIL, KGKTMKADPVGAQEY, and KPKGKTMKADPVGAQ), four epitopes of *Ov-CF-1* (ELRFRIFKDNLERAK, YEEFKLKYKKTYSND, DEPIVNEDPTPQEDV, QSKFVAYVNGSTRLP) and two epitopes of *Ov-CALR* (SNPDYQPDPDLYVRD, PDNKFKVLIDNEQVE) for the formation of a final vaccine construct. All MHC2 epitopes and their antigenicity analysis are listed in **Supplementary Tables 4**–**6**.

### B-cell epitope prediction

B cells are immune cells that give rise to specific immunoglobulins when they come in contact with antigens or foreign particles. Selection of appropriate B-cell epitopes is crucial in designing multi-epitope vaccines as they can stimulate different immunogenic mechanisms, e.g., neutralization of antigens, complement system activation, and cytotoxicity by T cells (58). We determined B-cell epitopes of the selected target proteins by using the BepiPred Linear Epitope Prediction 2.0 method from IEDB. From the output dataset of epitopes, we selected a total of 11 B-cell epitopes ranging in length from 10 to 40 amino acids. Next, we performed antigenic and allergenicity analyses of chosen epitopes that further narrowed down the number of epitopes to four from *Ov-TPx-1* (VYAHLQWTKMDRKAGGLGK, VVNGEFKNISLKDYR, TVNDRPVGRSVEEA, WKPKGKTMKADPVGAQE) based on high antigenicity and non-allergenicity **(Supplementary Table 7**). While none of the B cell epitopes were selected from *Ov-CF-1* and *Ov-CALR* because of their low antigenicity from the default threshold (0.5) and role in allergenicity. The final B-cell epitopes are given in **Supplementary Table 7**.

### Vaccine construction

**Figure 2:**
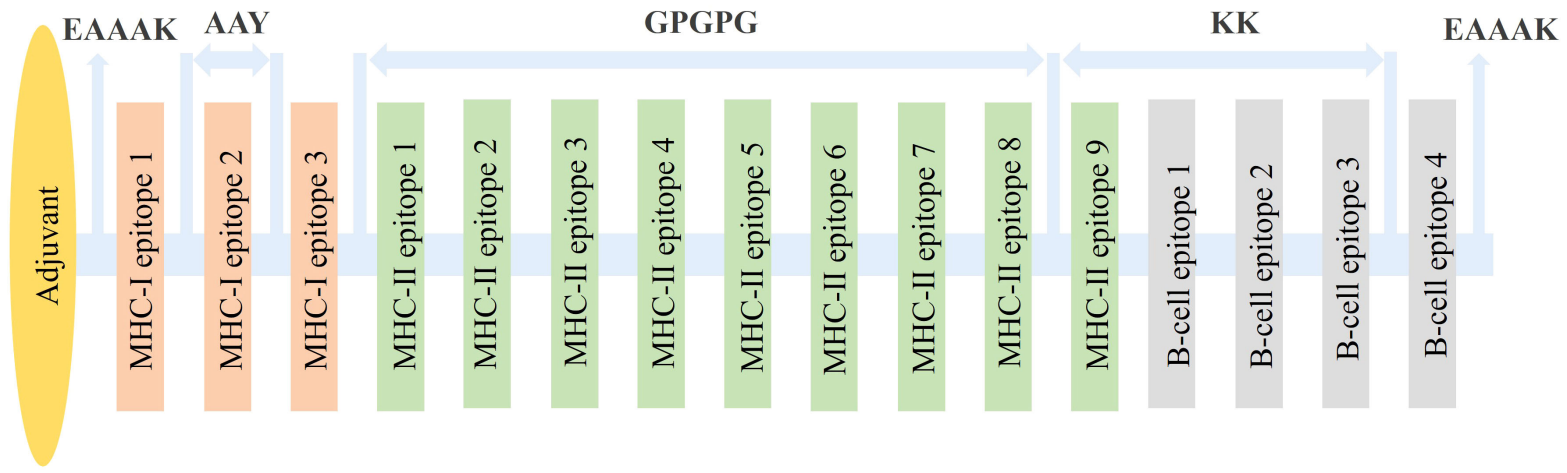
Vaccine construct designed with epitopes and adjuvant linked through different linkers.

Adjuvants and linkers are used in vaccine construction as they enhance the durability, immunogenicity, and efficiency of the vaccine. In this study, we designed four vaccine constructs (**Table 2**) by adding four different adjuvants at the C-terminals. The adjuvants used in this study thewere ribosomal protein adjuvant, heparin-binding hemagglutinin, granulocyte-macrophage colony, and HBHA adjuvant. Different linkers, e.g., EAAAK, AAY, GPGPG, and KK, were used to join adjuvants, CTL, HTL, and B-cell epitopes, respectively, in vaccine formation (**Figure 2**). Further, the designed vaccines were evaluated for their toxin, antigenic, and allergenic properties. Vaccines were found to be non-toxin, antigenic, and non-allergenic in nature.

**Table 2:** Final sequence of designed vaccine with the adjuvants used to design them.

Vaccine construct	Adjuvant	Designed vaccine sequence
1	L7/L12 Ribosomal protein adjuvant	MAKLSTDELLDAFKEMTLLELSDFVKKFEETFEVTAAAPVAVAAAGAAPAGAAVEAAEEQSEFDVILEAAGDKKIGVIKVVREIVSGLGLKEAKDLVDGAPKPLLEKVAKEAADEAKAKLEAAGATVTVKEAAAKALYEEFKLKAAYLFYPLDFTFAAYEEGHAFRGQFGPGPGSNPDYQPDPDLYVRDGPGPGPDNKFKVLIDNEQVEGPGPGELRFRIFKDNLERAKGPGPGYEEFKLKYKKTYSNDGPGPGDEPIVNEDPTPQEDVGPGPGQSKFVAYVNGSTRLPGPGPGKNISLKDYRGKYVILGPGPGKGKTMKADPVGAQEYGPGPGKPKGKTMKADPVGAQKKVVNGEFKNISLKDYRKKVYAHLQWTKMDRKAGGLGKKKTVNDRPVGRSVEEAKKWKPKGKTMKADPVGAQEEAAAK
2	Heparin-binding hemagglutinin	MAENSNIDDIKAPLLAALGAADLALATVNELITNLRERAEETRTDTRSRVEESRARLTKLQEDLPEQLTELREKFTAEELRKAAEGYLEAATSRYNELVERGEAALERLRSQQSFEEVSARAEGYVDQAVELTQEALGTVASQTRAVGERAAKLVGIELPKKAAPAKKAAPAKKAAPAKKAAAKKAPAKKAAAKKVTQKEAAAKALYEEFKLKAAYLFYPLDFTFAAYEEGHAFRGQFGPGPGSNPDYQPDPDLYVRDGPGPGPDNKFKVLIDNEQVEGPGPGELRFRIFKDNLERAKGPGPGYEEFKLKYKKTYSNDGPGPGDEPIVNEDPTPQEDVGPGPGQSKFVAYVNGSTRLPGPGPGKNISLKDYRGKYVILGPGPGKGKTMKADPVGAQEYGPGPGKPKGKTMKADPVGAQKKVVNGEFKNISLKDYRKKVYAHLQWTKMDRKAGGLGKKKTVNDRPVGRSVEEAKKWKPKGKTMKADPVGAQEEAAAK
3	HBHA Adjuvant	MAENPNIDDLPAPLLAALGAADLALATVNDLIANLRERAEETRAETRTRVEERRARLTKFQEDLPEQFIELRDKFTTEELRKAAEGYLEAATNRYNELVERGEAALQRLRSQTAFEDASARAEGYVDQAVELTQEALGTVASQTRAVGERAAKLVGIELEAAAKALYEEFKLKAAYLFYPLDFTFAAYEEGHAFRGQFGPGPGSNPDYQPDPDLYVRDGPGPGPDNKFKVLIDNEQVEGPGPGELRFRIFKDNLERAKGPGPGYEEFKLKYKKTYSNDGPGPGDEPIVNEDPTPQEDVGPGPGQSKFVAYVNGSTRLPGPGPGKNISLKDYRGKYVILGPGPGKGKTMKADPVGAQEYGPGPGKPKGKTMKADPVGAQKKVVNGEFKNISLKDYRKKVYAHLQWTKMDRKAGGLGKKKTVNDRPVGRSVEEAKKWKPKGKTMKADPVGAQEEAAAK
4	Granulocyte-macrophage colony	MWLQSLLLLGTVACSISAPARSPSPSTQPWEHVNAIQEARRLLNLSRDTAAEMNETVEVISEMFDQEPTCLQTRLELYKQGLRGSLTKLKGPLTMMASHYKQHCPPTPETSCATQIITFESFKENLKDFLLVIPFDCWEPVQEEAAAKALYEEFKLKAAYLFYPLDFTFAAYEEGHAFRGQFGPGPGSNPDYQPDPDLYVRDGPGPGPDNKFKVLIDNEQVEGPGPGELRFRIFKDNLERAKGPGPGYEEFKLKYKKTYSNDGPGPGDEPIVNEDPTPQEDVGPGPGQSKFVAYVNGSTRLPGPGPGKNISLKDYRGKYVILGPGPGKGKTMKADPVGAQEYGPGPGKPKGKTMKADPVGAQKKVVNGEFKNISLKDYRKKVYAHLQWTKMDRKAGGLGKKKTVNDRPVGRSVEEAKKWKPKGKTMKADPVGAQEEAAAK

### Physicochemical property of vaccine

The theoretical Pi, aliphatic index, molecular weight, instability index, and GRAVY score of the constructed vaccine were determined by the use of the ProtParam Expasy server. The molecular weights of the vaccine constructs ranged from 45 to 53 kda indicating that all vaccines were active antigens with a weight greater than 8 Kb. The vaccines have a basic nature, as the theoretical Pi value falls between 8.23 and 9.38. All the vaccines were stable *in vitro* because their instability index was found to be less than 40, which is an ideal value for antigen stability. At the natural temperature of the body, the aliphatic index ranged from 62 to 67, which indicated the thermostability of the vaccine at body temperature. The negative GRAVY value ranging from 0.6 to 0.82 proved that the vaccines are hydrophilic and can interact with the surrounding water molecules. Solpro Server was used for solubility evaluation of vaccine constructs, and its results ranging from 0.60 to 0.87 (more than threshold 0.5 for soluble vaccines) showed that designed vaccines are soluble in nature. VaxiJen 2.0 and the AntigenPro server were used to check the antigenic properties, and it was found that the vaccines are potential antigens. AllerTop v2.0 evaluated the non-allergen properties of vaccines. The physicochemical properties suggest that all vaccines are highly efficient, thermostable, non-antigens, and non-allergens (**Table 3**).

**Table 3:** Physicochemical properties analysis of designed vaccine constructs.

Physicochemical parameters	V1		V2		V3		V4	
	Measurement	Indication	Measurement	Indication	Measurement	Indication	Measurement	Indication
Molecular weight	45522.84	Appropriate	53616.82	Appropriate	49711	Appropriate	48263.97	Appropriate
Number of amino acids	425	Appropriate	494	Appropriate	454	Appropriate	438	Appropriate
Nucleotides	1275	Appropriate	1482	Appropriate	1362	Appropriate	1314	Appropriate
Solubility by Prot-Sol	0.658	Soluble	0.701	Soluble	0.596	Soluble	0.417	Soluble
Solubility by Sol-pro	0.73793	Soluble	0.865411	Soluble	0.8022	Soluble	0.6173	Soluble
Aliphatic index	67.55	Thermostable	64.92	Thermostable	65.42	Thermostable	62.15	Thermostable
Instability index	18.69	Stable	29.64	Stable	27.11	Stable	34.82	Stable
Antigenicity by VaxiJen	0.5693	Antigenic	0.6601	Antigenic	0.6997	Antigenic	0.6887	Antigenic
Antigenicity by ANTIGENPro	0.919299	Antigenic	0.929111	Antigenic	0.9081	Antigenic	0.9156	Antigenic
Theoretical PI	8.48	Basic	9.37	Basic	8.24	Basic	8.83	Basic
Allergenicity	Non-allergen	Non-allergenic	Non-allergen	Non-allergenic	Non-allergen	Non-allergenic	Non-allergen	Non-allergenic
Grand average of hydropathicity (GRAVY)	-0.62	Hydrophilic	-0.819	Hydrophilic	-0.806	Hydrophilic	-0.734	Hydrophilic

### Population coverage

**Figure 3:**
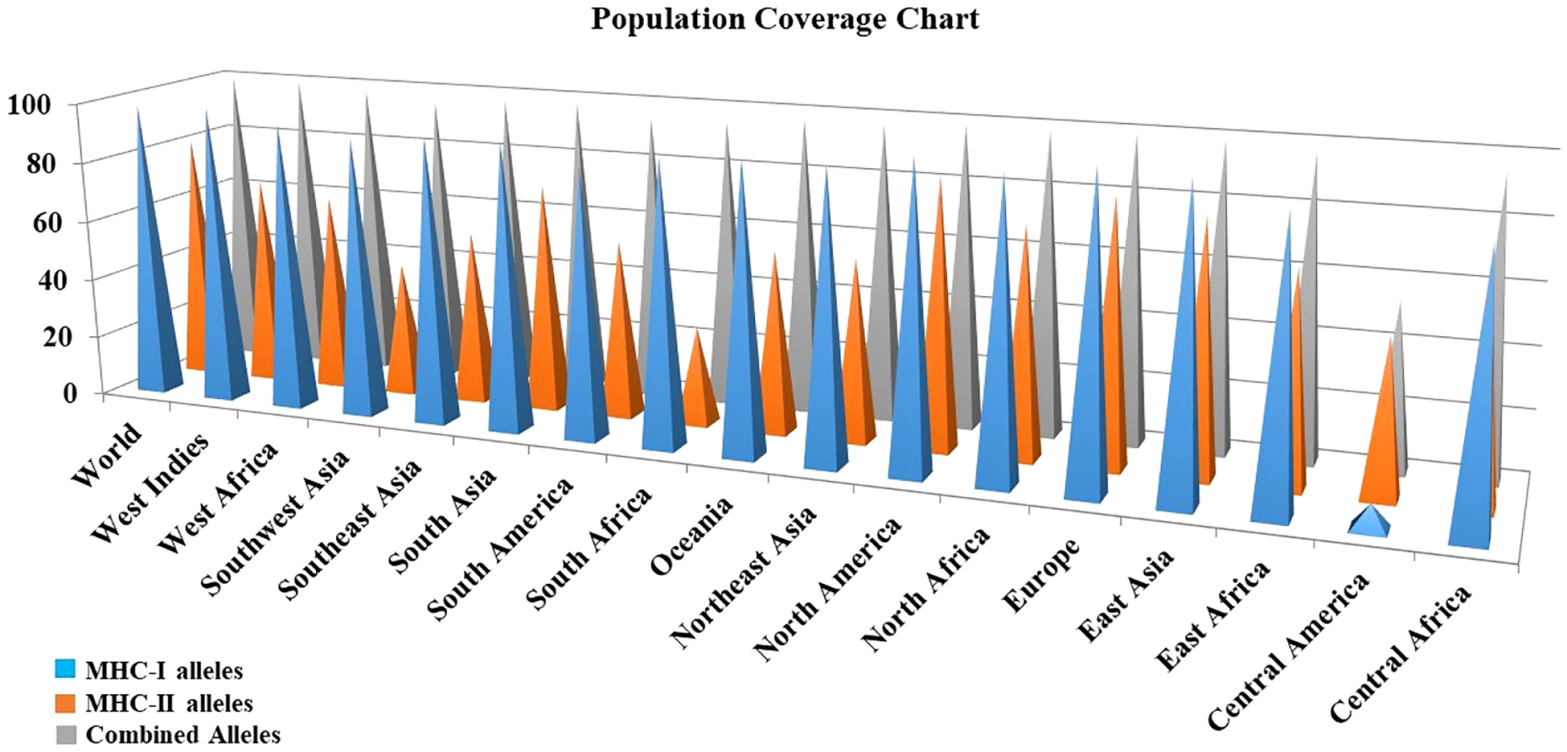
Population coverage analysis of the prioritized MHC epitopes selected for vaccine construction.

Due to regional and ethnic variations, there may be slight differences in the expression of HLA alleles worldwide. Furthermore, a detailed analysis of the distribution of HLA alleles across the global population is essential for the successful production of vaccines. For population coverage analysis, specific CTL and HTL epitopes that were used in the vaccine’s construction as well as the related HLA alleles were acquired from this analysis. The statistical results showed 98.55%, 81.81% and 99.77% world population coverage for MHC-1, MHC-2 and combined HLA alleles respectively as given in **Figure 3**.

### Vaccine’s structure analysis

**Figure 4:**
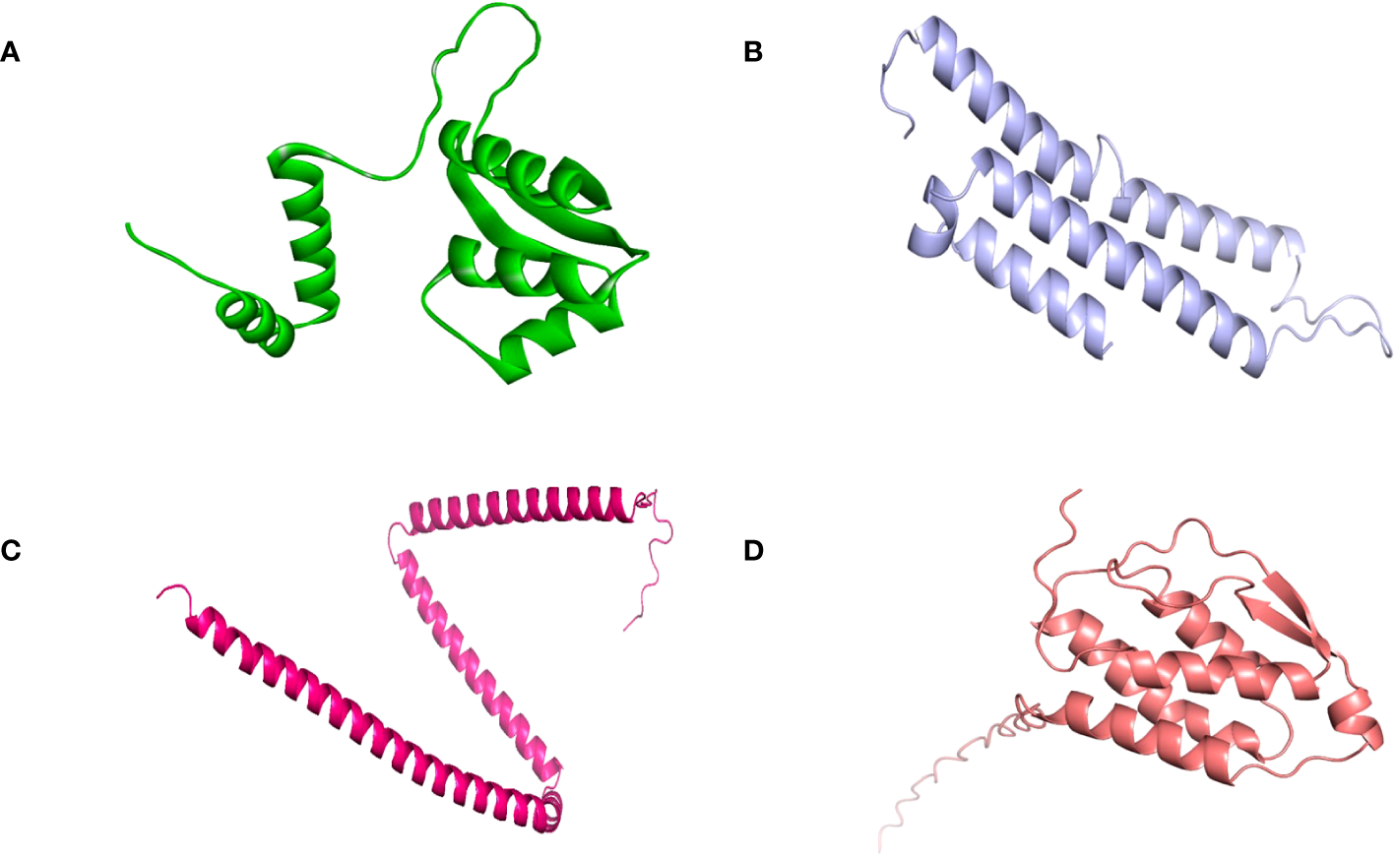
Tertiary structure of the vaccine constructs. **(A)** Vaccine-1 **(B)** Vaccine-2 **(C)** Vaccine-3 **(D)** Vaccine-4.

**Figure 5:**
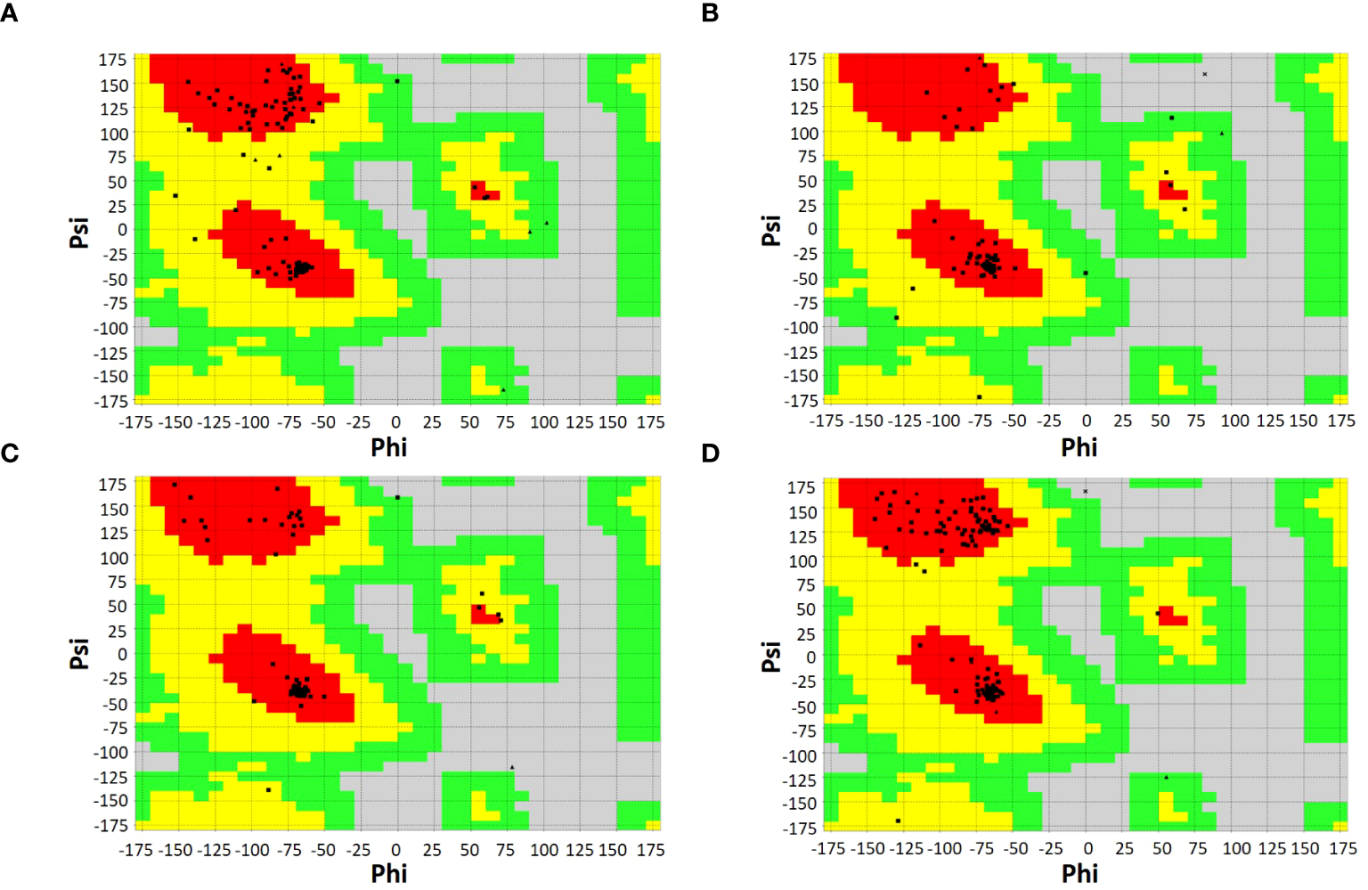
Ramachandran plots of vaccine constructs showing >90% residues in most favorable region (red), favorable region (yellow), and non-favorable region (grey). **(A)** V1 (96.6%) **(B)** V2 (92.8%) **(C)** V3 (98.1%) **(D)** V4 (96.8%).

**Figure 6:**
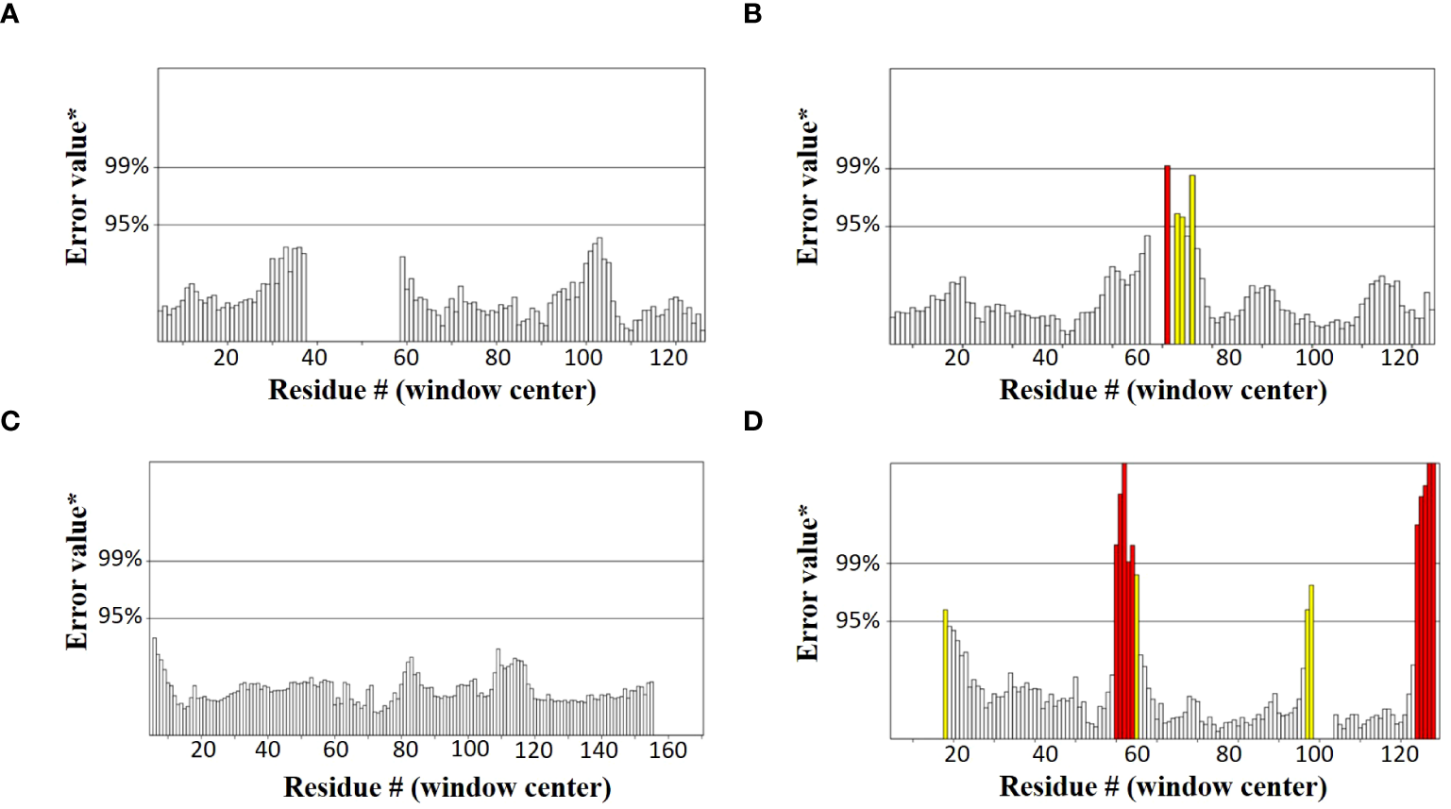
Graphs showing ERRAT values of constructed vaccines **(A)** V1 (100) **(B)** V2 (96.10) **(C)** V3 (100) **(D)** V4 (87.93).

SOPMA and PSIPRED servers were used for the prediction of the secondary structure of the vaccine constructs. The results showed different percentages of α-helix, random coil, and β-strand structures in all four constructed vaccines without any error (**Supplementary Figure 1**). The 3D structures of the vaccines were predicted using the Swiss Model server (**Figure 4**). GalaxyRefine server was used for the refinement and improvement of the quality of the vaccine 3D models. The quality inspection and validation of all models was carried out by ERRAT values in SAVES server v6.0 and Ramachandran plot analysis in Vadar server to elect the top models having a greater percentage number of favorable regions residues (**Figure 5**). ERRAT values, i.e., 100, 96.10, 100, and 87.93 of V1, V2, V3, and V4, respectively, have shown that there are minimum errors in protein folding and configuration because the more is the ERRAT score, the higher is the quality of the designed vaccines (**Figure 6**) (59). The results of the Ramachandran plot indicated 96.6%, 92.8%, 98.1%, and 96.8% residues in the favored region for the four vaccines. As an ideal model has >90% favored region residues, this suggests that all vaccine models are capable of protein-protein docking and molecular dynamic simulation analysis.

### Conformational B-cell epitopes prediction

The conformational B-cell epitopes are exposed to surface and are easily accessible to the solvent. They are generally formed by protein folding and are easily accessible to B-cells and antibodies; therefore, they help in the induction of a potent immune response. With scores greater than 0.7 on the ElliPro server, a total of 45, 33, 49, and 44 residues were identified to be present in the conformational B cell epitopes of V1 to 4, respectively (**Supplementary Figure 2**). Residues involved in the conformational epitopes formation are given in **Supplementary Table 8**.

### Molecular docking and binding affinity evaluation

**Figure 7:**
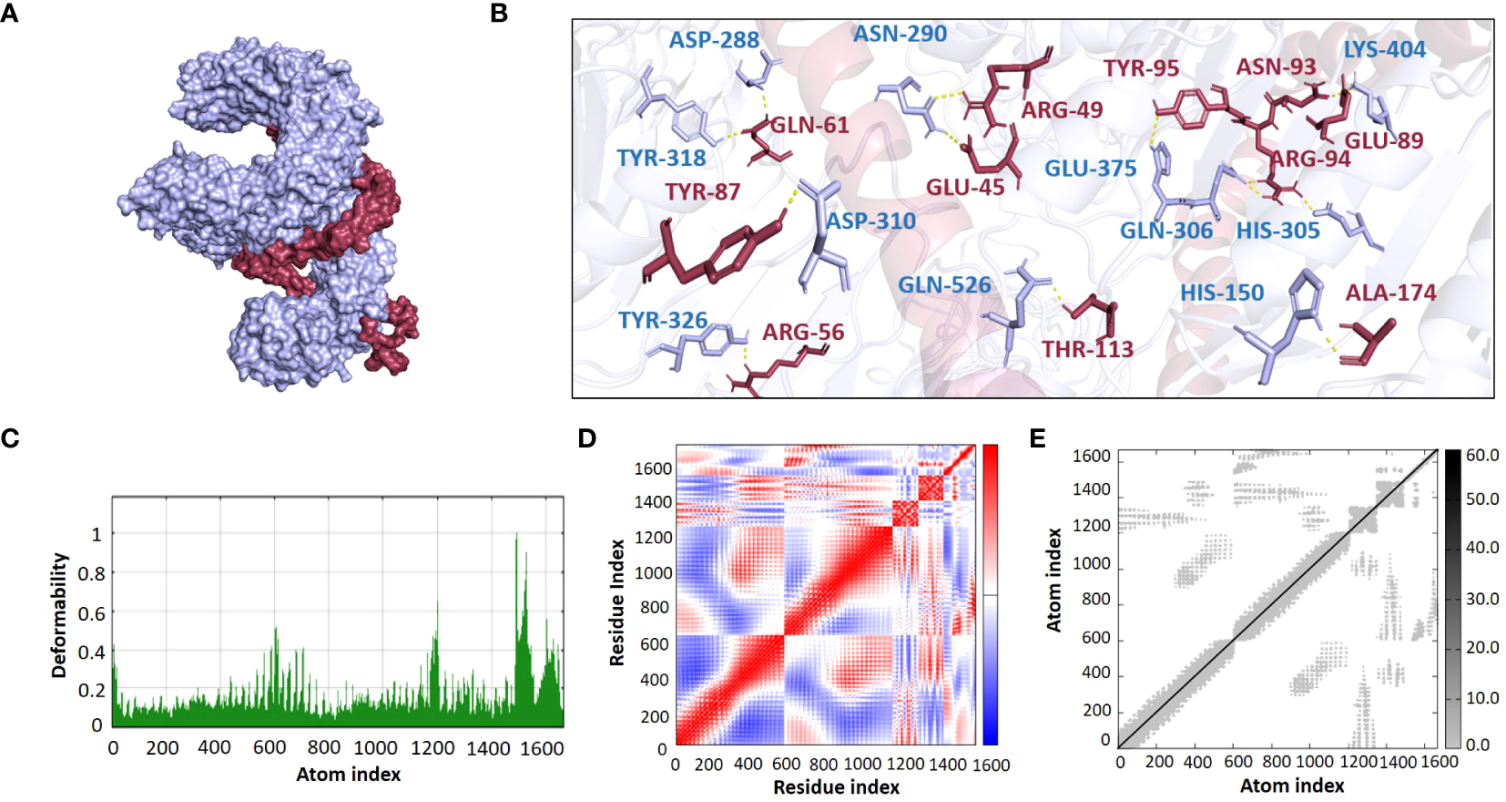
**(A)** Visualization of V3-TLR2 docked complex **(B)** Molecular Interactions of Vaccine V3 (raspberry) with TLR2 (light blue) **(C)** Deformability **(D)** Covariance **(E)** Elastic Network Model.

**Figure 8:**
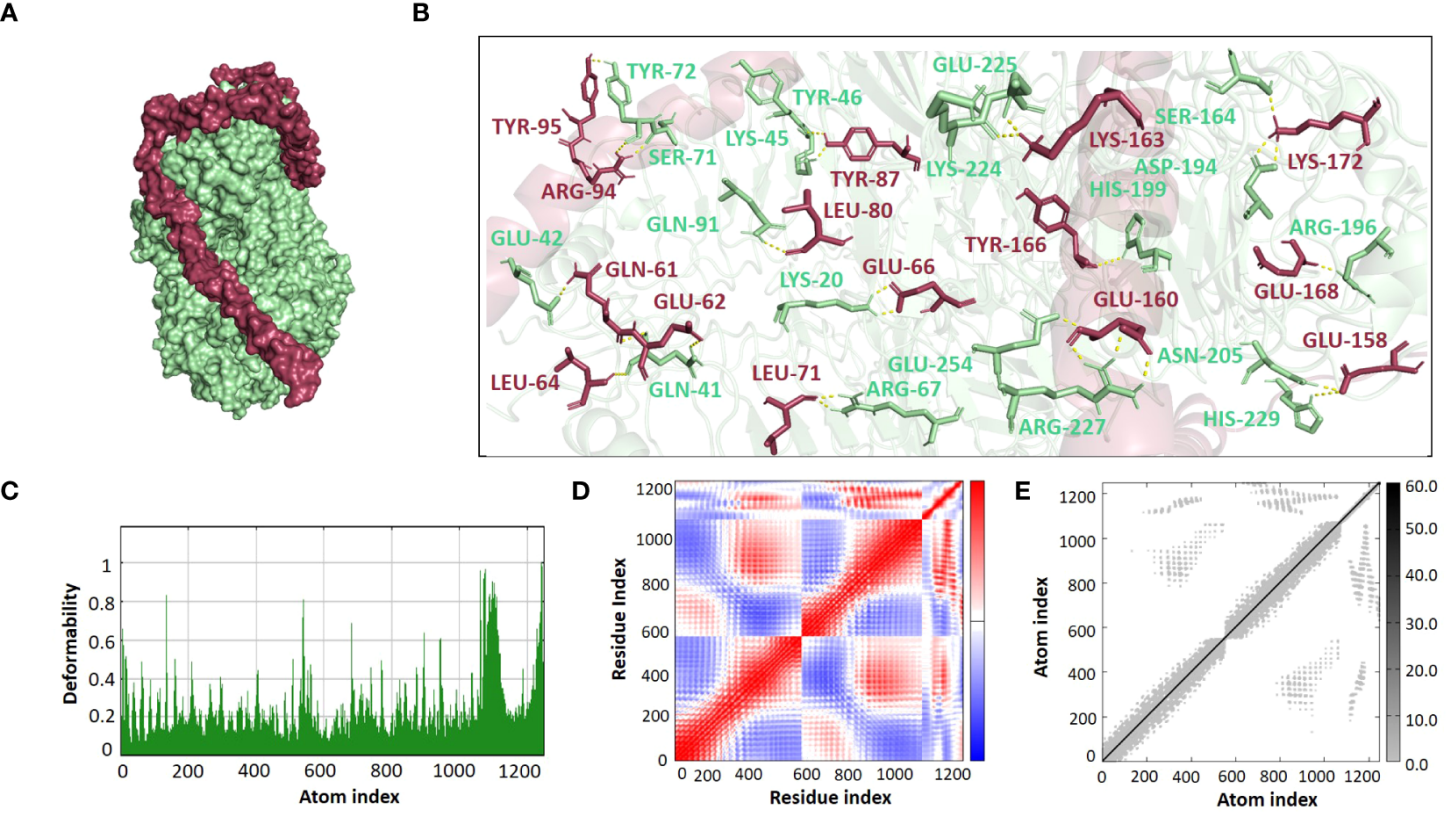
**(A)** Visualization of V3-TLR4 docked complex **(B)** Molecular Interactions of Vaccine V3 (raspberry) with TLR4 (light green) **(C)** Deformability **(D)** Covariance **(E)** Elastic Network Model.

The ClusPro 2.0 webserver was used to predict the interaction between the designed vaccine 3D models and immune receptors. It is necessary to prepare the vaccines and the receptors before docking in order to remove the extra chains and water molecules. We prepared our vaccines and receptors by using the Molecular Operating Environment (MOE) software. The prepared vaccines were then docked to prepared human immune receptors, namely TLR-2 (ID: 2Z7X) and TLR-4 (ID: 3FXI). The server provided the top ten complexes for each vaccine with their respective binding energies. For each vaccine, one model was selected with the lowest binding energy because low scores depict a high binding tendency of vaccines with immune receptors. The docking scores of the V1, V2, V3, and V4 were found to be -822.9, -742.9, -1219.1, and -1348.4, respectively, with the receptor TLR2, while with TLR4, docking scores were found to be -904.9, -882.3, -1172.6, and-1320.3, respectively. Determination of the interactions between vaccine constructs (V1–4) and toll-like receptors (TLR) 2 and 4 yielded important information about the ramifications for immune response modulation and vaccine design. We observed certain receptor residues and vaccines with significant interactions by examining the interactions of vaccine models with TLR2 and TLR4 receptors (**Table 4**). The resulting docking scores of vaccines in complex with TLR-2 and TLR-4 showed that constructs 3 and 4 have the lowest binding energy for both receptors. These two vaccines were then compared on the basis of their interactions with the receptors which concluded that V3 has the highest number of interactions as well as lowest binding energy. The interactions of the V3 docked complexes are shown in **Figures 7**, **8**. Therefore, V3-TLR2 & V3-TLR4 docked complexes were further employed for MD simulation analysis to check their binding affinity and stability.

**Table 4:** Molecular interactions between vaccine constructs (V1, V2, V3, and V4) and Toll-like Receptors TLR-2 and 4.

Vaccine construct	Interacting Residues	Receptor	Interacting Residues
V1	A45, A47, GL55, A53, A50, PR49, GL51, A48, V54.	TLR2	G285, ASP310, TY326, PH325, VA339, GL316, TR258, G286, P12, V311,
V1	G115, G83, PH32, VA85, S86, GL106, LY107, TH35, ME1, AL2, L3, G8, G15, GL20, GL106, LY107.	TLR4	GLN599, AR598, GL562, S622, AS624, IL625, GL588, S589, GL510, G509, AS580, LY560, GL562, AS624, AR598, H587.
V2	AR36, GL113, G70, AS22.	TLR2	S109, S63, AR80, AR80, LY104
V2	AR36, S114, GL117, AL120, G113, AR108, GL67, G70, LY74, GL89, GL85, G86, AL83, LY82, AR81.	TLR4	AS596, AR598, GL599, G547, G523, GL605, S570, GL430, AR382, AS379, AR355, GLU336, ARG96.
V3	L302, TY87, GL89, GL61, AR56, AR49, G45, IL211, AS93, ARG94, TY95.	TLR2	H150, LE302, LY404, GLU375, AS310, TY318, AS238, TY326, AS290.
V3	A94, T87, L80, L71, G61, L64, G66, L172, G158, G160, L163, T166, G168, L172,G61, L64, G66, L71.	TLR4	H229, A205, A227, G225, H199, A196, A194, S164, G42, G91, A67, L47, S71, T72, T46, L224, G41, L20
V4	P322, A305, P206, L308, A337.	TLR2	M1, L9, V12, T11
V4	A496, A468, S441, A464, S127, A83.	TLR4	C14, T11, G10, V12, L9.

### Normal mode analysis

The binding affinities of the best-docked complexes of V3 with the immune receptors (TLR2 and TLR4) were validated using MD analysis through the iMODS server (**Figures 7**, **8**), which executed Normal Mode Analysis for complex deformability, eigenvalue, variance, covariance, elastic network model, and B-factor evaluation of complexes. The deformability of a complex is its ability to deform at its residues. The hinges in the graph represent areas of high deformability (**Figures 7C**, **8C**). Eigenvalue indicates the motion stiffness of the complex. A lower eigenvalue means that it is easier to deform the structure, as it is in direct relation to the energy needed for deformation. The eigenvalues of V3 complexed with TLR2 and TLR4 are 3.734039e-06 and 3.262975e-06, respectively. Each normal mode’s variance and eigenvalue were found to be negatively correlated. The covariance matrix indicated whether two residue pairs experienced correlated (red), uncorrelated (white), or anti-correlated motions (**Figures 7D**, **8D**). The elastic network concept determined the pairs of atoms connected by springs. In the graph, each pair of atoms was represented by a single spring, and the color of the dots indicated how stiff the spring was—darker grays denoted stiffer springs, and vice versa (**Figures 7E**, **8E**).

### Molecular dynamic simulations

**Figure 9:**
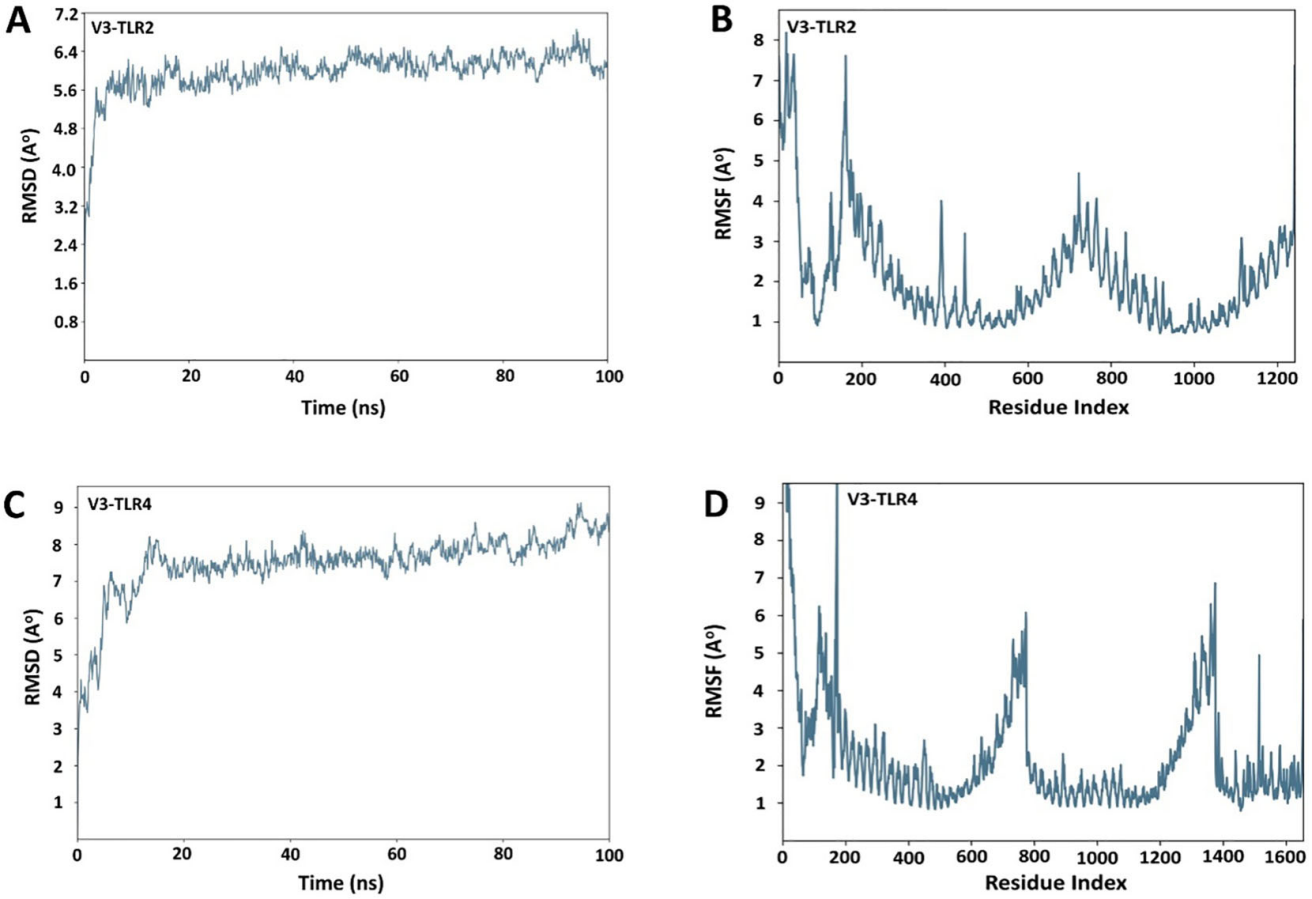
RMSD and RMSF plots for receptors and the vaccine construct complexes **(A)** RMSD plot of the V3-TLR2 complex **(B)** RMSF plot of the V3-TLR2 complex **(C)** RMSD plot of the V3-TLR4 complex **(D)** RMSF plot of the V3-TLR4 complex.

Desmond software from Schrödinger LLC was used for molecular dynamic simulation studies of the prioritized docked complexes (V3 in complex with TLR2 & TLR4). The simulation time for both complexes, V3-TLR2 & V3-TLR4, was set to 100 ns. The RMSD of both the complexes was calculated in the bound conformations of the receptors and vaccine. The RMSD profile of the V3-TLR2 complex showed stabilization after an initial equilibration phase, maintaining fluctuations between 5.6 and 6.4 Å throughout the 100 ns simulation (**Figure 9A**), indicating overall structural stability. RMSF analysis revealed moderate flexibility in V3-TLR2, mainly at the N terminal region (**Figure 9B**). The V3-TLR4 complex also remained stable, with RMSD values mostly between 7 and 8 Å and reaching ~ 9 Å in the end (**Figure 9C**), though it exhibited higher fluctuations during initial equilibration phase. While the RMSF analysis of V3-TLR4 showed more pronounced fluctuations at the N- and C-terminal regions (**Figure 9D**). In case of V3-TLR4 complex, the RMSD was calculated to be 11 Å ± 1 Å and this indicated some deviations in the initial state and a minor variation was observed between 2500 to 3500ns but later on, no significant deviations were observed (**Figure 9B**). The stability of the V3-TLR2 & V3-TLR4 complexes is confirmed by their RMSD plots. The presence of fundamentally flexible regions can impute the observed increased fluctuations in RMS values in both complexes. This is significantly complexed with the inclusion of loop regions including epitopes, linker sequences and adjuvant attached at the N-terminal of the designed vaccine construct. This substantial fluctuation tendency is consistent with previous study findings and lends support to the notion of structural dynamics in this setting (57). RMSF can be used to characterize local modifications along the protein chain. Peaks in the RMSF graphs represent areas of the protein that fluctuate the most during the simulations. As a result, a residual flexibility study was performed to better evaluate the stability of the generated complexes, which revealed that both immune receptors have reduced flexibility values (**Figures 9B, D**), confirming our findings.

### Codon adaptation and cloning

Codon adaptation can be defined as a technique that makes codons more effective for bacterial genomes, leading to a higher expression rate. Since humans and the intended host use different codons, this method was used to speed up the synthesis of vaccines in the K12 strain system of *E. coli*. To achieve a high expression of proteins, the Java codon adaptation tool was used to calculate the ideal CAI value and GC content. In this study, after optimization of sequences, the CAI value generated was 0.98 and GC content percentage was 52.42% for V3. The absolute GC content scopes in between 30% and 70% (60), and a CAI value of 0.8 is typically regarded as a high score (61). These results are consistent with earlier researches showing same range of data necessary for durable vaccine expression and validate the higher expression levels for the prioritized vaccine (33). Finally, by using the SnapGene tool, *in silico* clone of optimized sequence of V3 was made by inserting sequences in the pET28a (+) vector at the PshA1 restriction site (**Supplementary Figure 3**), resulting in a final genome size of 6731 bps.

### Immune response analysis

**Figure 10:**
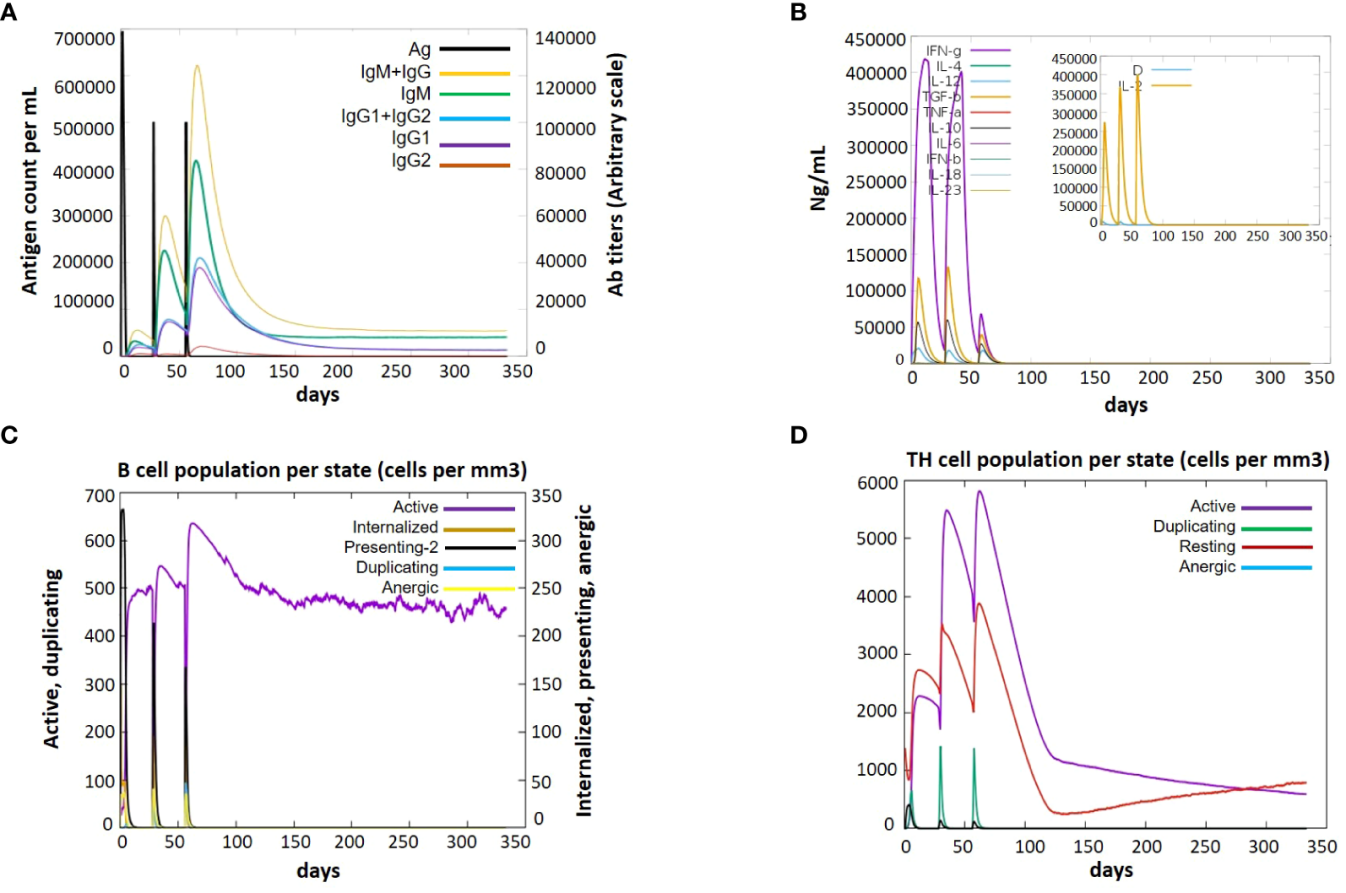
Immune response after administration of V3 to the host achieved via Immune Simulations in three doses (1, 84 and 168 hr) **(A)** Antigen Count **(B)** Cytokine Induction **(C)** B-cell population per state **(D)** TH population per state.

Immune simulation is essential for the development of vaccines. An immunologic response that is a defending mechanism against a specific pathogen is intended to be produced by a vaccination, which has no harmful side effects. Through the use of indicators such as antibodies, cytokines, B and T cells, and NK cells, the framing of a fruitful vaccine and its immunologic responses are assessed. To evaluate the immunological response initiated by our vaccine-3, we employed immune simulations. The immune responses were boosted by the addition of each booster dose. A sudden increase in cytokine levels was also seen with each dose, with comparable amounts. Notably, the vaccine induced the production of cytokines like TGF-b, IL-2, IL-18, IL-10, and IFN-g. The V3 generated a variety of targeted antibodies, including IgM, IgG, IgG1, IgG2. Each dose showed a dramatic drop in the antigen and an increase in the frequency of antibodies. After vaccination, there were many activated B cells, which produced plasma cells that are in charge of manufacturing certain antibodies. After immunization, there was also a noticeable increase in B-cell duplication. Additionally, the T-cell number increased following vaccine dose, with a sharp rise in activated T-cells, and a matching drop in inactive cells, demonstrating that the cells are activated after vaccination (**Figure 10**).

### Vaccine’s safety analysis

pBLAST was performed against *Homo sapiens* proteins to verify the safety profile of the developed vaccine. It was found that our vaccine showed no homology with any human protein. This indicated that our vaccine might be completely safe to use and there will be no possibility of an auto-immune response following vaccination.

## Discussion

Cholangiocarcinoma, caused by *O. viverrini*, is a serious disease because of its dreadful effects on human life, with a less than 5% survival rate within 5 years after diagnosis (62). The country with the highest rate of cholangiocarcinoma (CCA) is Thailand, with an estimated annual cost of US$120 million in medical care and lost wages due to opisthorchiasis and CCA (63) *O. viverrini* adopts multifactorial mechanisms, including the release of excretory-secretory products (ESPs) containing oncogenic proteins, involved in inflammation and the development of biliary cancer (13, 64). The only drug targeting *O. viverrini* is Praziquantel, which is effective against a slight infection but has many side effects and results in recurrent infections leading to CCA (14, 65). Currently, there is no effective drug or vaccine against opisthorchiasis-related CCA. Researchers are looking into vaccine therapy as a viable remedy because there aren’t many medicinal options (18).

The goal of the current study is to frame an efficient vaccination against *O. viverrini*. To boost the immune response against *O. viverrini*, we developed effective constructs were developed using a multi-epitope method by choosing epitopes from tumor antigens. In this study, we selected thioredoxin peroxidase (*Ov-TPx-1*), cathepsin F (*Ov-CF-1*) and calreticulin (*Ov-CALR*) proteins of *O. viverrini* were selected as literature has shown their role in inflammation and CCA development by antioxidant, ECM, and hemoglobin degradation, and host immune system modulating activities (66). Thioredoxin peroxidases (TPx) have antioxidant and anti-apoptotic potential for parasite survival and CCA growth. It shields the parasite from harm brought on by reactive oxygen species and inflammation (67–69). Cathepsin F1, a cysteine protease, is secreted by *O. viverrini* in the bile duct, and results in the degradation of hemoglobin and extracellular matrix (ECM) proteins, thus accelerating hepatobiliary pathologies and cholangiocarcinoma. It plays a role in immune invasion, nutrition, and host-parasite relationships (70, 71). Calreticulin of *O. viverrini* (*Ov CALR*) is important for parasite fecundity and modulates the host immune system by binding with the C1q polypeptide of the complement system, affecting its role in inflammation and tumor cell attacks. It has both cancer-supporting and cancer-inhibiting potential for CCA development (23). Inhibition of these proteins may improve the host’s capacity to produce an adequate immune-mediated response, leading to elevate infection control or clearance. By targeting a key enzyme which is essential for the survival of pathogen, could offer an effective approach for treating infection. Therefore, these proteins were selected for epitope prediction.

Epitopes are the smallest immunogenic peptides within an antigen that can trigger either helper or cytotoxic T-cells (72). T cell-binding epitopes are necessary for multi-epitope vaccines because they make it easier for HTLs and CTLs to recognize the epitopes, which are necessary for specific immunoglobulins production and pathogen eradication, respectively.

The T- and B-cell epitopes were identified by various methods and were joined using linkers and adjuvants for the vaccine construction. MHC-I, MHC-II, and B-cell epitopes were joined using the Ala-Ala-Tyr (AAY), Gly-Pro-Gly-Pro-Gly (GPGPG), and bi-lysine (KK) linkers. The immunogenicity of the designed vaccines is increased by these immunogenic linkers. In addition, linkers assist in maintaining the separation of epitopes and inhibit their folding (73, 74). The AAY linker is a common type of linker used to connect two protein domains. It has been shown that by increasing the access of the immune cells to the antigenic sites, this linker enhances the immune response to vaccine antigens. It also preserves the structural integrity of the vaccine’s antigens (75). The GPGPG linker is another widely used linker in vaccine design. This linker’s flexibility makes it easy to fold the vaccine’s antigens. It also helps to lessen antigen aggregation, which can jeopardize the stability and immunogenicity of the antigen (76). The KK linker is widely used to attach a protein antigen to a carrier protein, like a bacterial toxin. The positive charge of the linker stabilizes the interaction between the carrier protein and antigen, resulting in a stronger immune response (77). Furthermore, the fusion protein’s stiffness, stability, and bi-functional catalytic activity were all improved by the use of an empirical-helical linker, EAAAK (75).

Different adjuvants, namely L7/L12 ribosomal protein, heparin-binding hemagglutinin adhesion (HBHA), granulocyte-macrophage colony were added to boost the immunogenicity of the vaccine constructs. L7/L12 Ribosomal-protein, often referred to as TLR4 binder, was used as it has been shown in prior studies to generate a robust immune response when employed as an adjuvant in disorders like cancer, etc (78). The granulocyte macrophage colony was employed since it has been suggested as a possible adjuvant for vaccines and has demonstrated the ability to boost immunogenicity against a number of infectious illnesses (79). Mycobacterium tuberculosis’s heparin-binding hemagglutinin adhesion (HBHA), an efficient immunological adjuvant, was also used. In addition to its ability to elicit a potent Th1 cell immune response, HBHA is a key player in the development of multi-epitope vaccines for immunotherapy. Its potent immune potential has also drawn a lot of attention. It can stimulate the migration of DCs and encourage the expression of a range of surface molecules (like CD80, CD40, and CD86), MHC I and MHC II molecules, as well as inflammatory cytokines (like IL-1β, IL-12, IL-6, and TNF-α), in a TLR4-dependent manner (80).

The physicochemical properties of the designed vaccines demonstrated favorable properties, and it was proved that the potential vaccine constructs had sufficient antigenic properties to adequately elicit an immunogenic response. According to the evaluation report, from different servers, the vaccine constructs were highly soluble and more hydrophilic. In this investigation, we discovered that the vaccines’ instability index was within the normal range (below 40), showing that vaccines are stable inside the host body (81). Analysis of the vaccine structures indicated that there was no deformability in the secondary structures of vaccines. Moreover, the HLA alleles, which are varying in different ethnicities, maintain the response to T-cell epitopes. More HLA alleles should bind to the T-cell epitope in order to increase population coverage. To predict the allele distribution globally, we choose the MHC1 and MHC2 epitopes corresponding to their specific HLA alleles. The results demonstrated that the selected epitopes and each of their unique alleles span an appropriate range of global geographic locations that is 99.77%. Swiss servers designed 3D models of vaccines, which were further refined by utilizing the Galaxy web server. For quality inspection and validation of all refined models, the Ramachandran plot and ERRAT score were used. A group of antigen residues that are separated from one another in the main sequence but are brought together spatially as a result of polypeptide folding is known as a discontinuous or conformational epitope. It is also known that conformational epitopes make up the majority of B-cell epitopes (around 90%) (82). Therefore, the residues involved in the formation of conformational B-cell epitopes of the vaccines were also predicted.

To examine the role played by the vaccine candidates in triggering an innate immune response, protein-protein docking studies with the immunologic receptors TLR-4 and TLR-2 were carried out. Toll-like receptors, TLR-4 and TLR-2 are present on a variety of immune cells i.e. NK cells, macrophages and some cancer cells. They distinguish between pathogen-associated patterns (PAMPs), and damage-associated molecular patterns (DAMPs) which helps to activate the immune system. TLR-4 and TLR-2 have shown superior tumor antigen recognition patterns and the capacity to trigger innate and acquired immune responses in the ten types of TLRs (Toll-like receptors) (83). ClusPro 2.0 outputs demonstrated that V3 and 4 have a high affinity for immune receptors. NMA and molecular dynamic simulation was performed to validate binding affinity of the constructs with the immune cell receptors and demonstrate the greater stability of the V3-receptor complexes. The low energy required to distort the complex, as indicated by lower eigenvalues obtained from the NMA study, defines the better stability of V3 complexed with TLR2 and TLR4. The combined findings of deformability, eigenvalue, variance, covariance, elastic network model, and B-factor showed a considerable affinity among the immune receptors (TLR2 and TLR4) and vaccine models, supporting the prediction that supposed vaccines can elicit an innate immune response (84, 85).

The codon adaptation of vaccines was carried out to increase codon usage by the host organism, and both vaccines were introduced inside the pET28-a (+) vector to make their clones. Later, immune simulation was carried out by an online tool, C-ImmSim in order to learn about the roles of vaccines in initiating a passive immune response. According to server outputs, exposure to the vaccine can increase the host’s immunoglobulin response and prolong the persistence of memory B cells even after three doses of the vaccine. Furthermore, a significant amount of IFN-gamma confirms our prediction that vaccines can activate an innate immune response. Bioinformatics approaches validated that the designed multi-epitope vaccines are effective and can trigger both, innate and adaptive immune responses successfully to eliminate the *O. viverrini* parasite.

In order to manage antigenic complexity, this work presented an alternate vaccination strategy based on the multi-epitope assembly of the protein components of the *O. viverrini* genome. The anticipated vaccine is thought to be immunogenic and was advised based on immunoinformatics techniques, although it is unclear how much they will protect against *O. viverrini* infections. Immunoinformatics techniques are very helpful for doing *in-silico* research and can direct lab investigations, which helps to save costs and time. However, the subsequent stage involves doing *in-vitro* immunological tests to verify the anticipated vaccine, ascertain its immunogenicity, and moreover design challenge-protection preclinical investigations to ultimately certify these methods. Therefore, the final vaccine construct-3 can be forwarded to the wet lab for *in vitro* and *in vivo* experimentation, which can offer a promising treatment option for CCA.

## Conclusion

Due to the limited therapies available for the terrible and rapidly spreading malignancy among the people of Southeast Asia, the creation of an effective vaccine against the parasite-mediated cholangiocarcinoma causing *O. viverrini* parasite is urgently required. By using an in-silico vaccine designing strategy and the careful analysis of tiny epitopes generated from the target antigenic proteins, namely, thioredoxin peroxidase (*Ov-TPx-1*), cathepsin F (*Ov-CF-1*), and calreticulin (*Ov-CALR*), our current study demonstrates the development of efficient vaccines against *O. viverrini*. Using immuno-informatics methods, it was possible to forecast potential epitopes that could be combined to make the vaccine. The developed vaccine revealed substantial affinity for the immune receptors TLR2 and TLR4 and showed encouraging results in terms of its physiochemical and immunogenic capabilities. The study proposed a vaccine construct that may activate inborn and acquired immunologic responses, that can provide a favorable alternative treatment for CCA-causing parasites. To validate the conclusions of our findings, additional experiments will be needed.

## Data availability statement

The original contributions presented in the study are included in the article/**Supplementary Material**. Further inquiries can be directed to the corresponding authors.

## Author contributions

MoS: Conceptualization, Methodology, Resources, Supervision, Writing – review & editing. FS: Formal analysis, Investigation, Writing – original draft. AS: Formal analysis, Investigation, Visualization, Writing – original draft. TW: Formal analysis, Investigation, Methodology, Visualization, Writing – original draft. MuS: Formal analysis, Investigation, Writing – original draft. AP: Formal analysis, Investigation, Methodology, Visualization, Writing – original draft. NU: Validation, Writing – review & editing. AZ: Formal analysis, Investigation, Methodology, Visualization, Writing – original draft. UN: Methodology, Software, Writing – review & editing. SA: Investigation, Writing – review & editing. RU: Formal analysis, Investigation, Visualization, Writing – review & editing. EA: Data curation, Validation, Visualization, Writing – review & editing. SO: Conceptualization, Funding acquisition, Supervision, Visualization, Writing – review & editing.

## Supplementary material

Supplementary Figure 1 Secondary structure of the Vaccines [V1 **(A)**, V2 **(B)**, V3 **(C)**, V4 **(D)**] containing α-helix, random coil, and β-strand. Pink shows the α-helix, yellow the β-stands and grey the coil regions of the designed vaccines.

Supplementary Figure 2 Residues of vaccine involved in conformational B cell epitope formation. Vaccine 1 [**(A)** 0.76 **(B)** 0.777]; vaccine 2 [**(C)** 0.844 **(D)** 0.738]; vaccine 3 [**(E)** 0.848 **(F)** 0.796]; Vaccine 4 [**(G)** 0.837 **(H)** 0.709]

Supplementary Figure 3
*In-silico* cloning of the final prioritized vaccine V3 in the pet28-a(+) vector. The black color represents the plasmid sequence while the red color indicates the vaccine sequence.
