# Correction: Development of a subunit vaccine against the cholangiocarcinoma causing *Opisthorchis viverrini*: a computational approach

**DOI:** 10.3389/fimmu.2025.1638914

**Published:** 2025-06-30

**Authors:** Mohibullah Shah, Farva Sitara, Asifa Sarfraz, Muhammad Shehroz, Tehreem Ul Wara, Asia Perveen, Najeeb Ullah, Aqal Zaman, Umar Nishan, Sarfraz Ahmed, Riaz Ullah, Essam A. Ali, Suvash Chandra Ojha

**Affiliations:** ^1^ Department of Biochemistry, Bahauddin Zakariya University, Multan, Pakistan; ^2^ Department of Bioinformatics, Kohsar University Murree, Murree, Pakistan; ^3^ Department of Microbiology & Molecular Genetics, Bahauddin Zakariya University, Multan, Pakistan; ^4^ Department of Chemistry, Kohat University of Science & Technology, Kohat, Pakistan; ^5^ Wellman Center for Photomedicine, Harvard Medical School, Massachusetts General Hospital, Boston, MA, United States; ^6^ Department of Pharmacognosy, College of Pharmacy, King Saud University Riyadh, Riyadh, Saudi Arabia; ^7^ Department of Pharmaceutical Chemistry, College of Pharmacy, King Saud University, Riyadh, Saudi Arabia; ^8^ Department of Infectious Diseases, The Affiliated Hospital of Southwest Medical University, Luzhou, China

**Keywords:** *Opisthorchis viverrini*, cholangiocarcinoma, epitope, vaccine, parasite; immunoinformatics-based vaccine designing

In the published article, there was an error in **Figure 9** as published. We identified that an incorrect figure was included due to its close resemblance to another dataset, as the MD simulations for both datasets were run on the same system using the same program and parameters. The corrected **Figure 9** and its caption “RMSD and RMSF plots for receptors and the vaccine construct complexes (A) RMSD plot of the V3-TLR2 complex (B) RMSF plot of the V3-TLR2 complex (C) RMSD plot of the V3-TLR4 complex (D) RMSF plot of the V3-TLR4 complex”. appear below.

**Figure 9:**
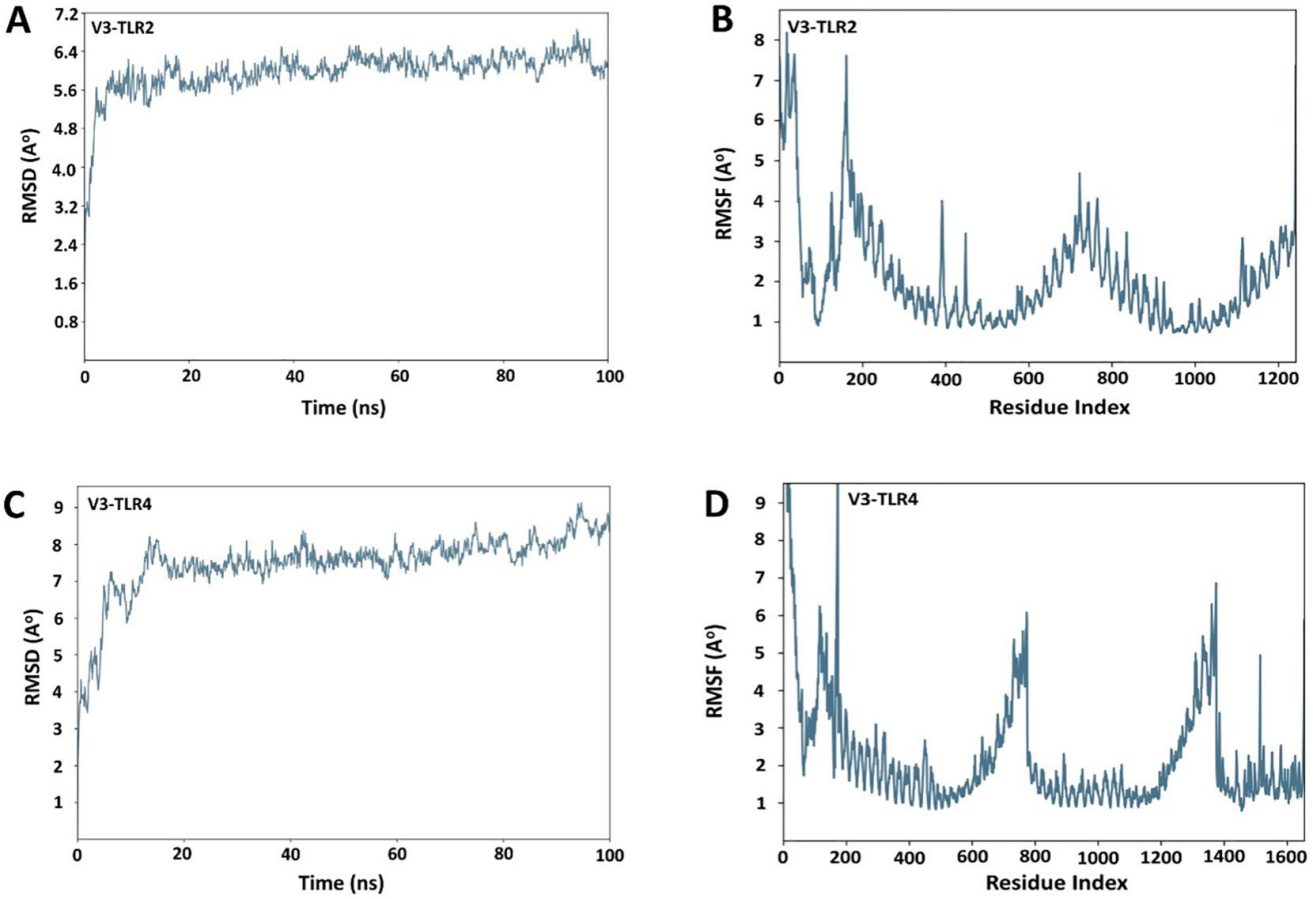
RMSD and RMSF plots for receptors and the vaccine construct complexes **(A)** RMSD plot of the V3-TLR2 complex **(B)** RMSF plot of the V3-TLR2 complex **(C)** RMSD plot of the V3-TLR4 complex **(D)** RMSF plot of the V3-TLR4 complex.

In the published article, there was an error. Due to the inclusion of an incorrect **Figure 9**, the corresponding text in the Results section also contains minor errors and typographic mistakes.

A correction has been made to **Results**, *Molecular dynamic simulations*, and paragraph 1. This sentence previously stated:

“The RMSD of both the complexes was calculated both in the unbound and bound conformations of the receptors and the vaccine. In **Figures 9A**, **C**, the RMSD was displayed as a histogram against the Cα atoms to evaluate the dynamic properties and conformational stability from initial to the final configuration of the protein. Minor departures from the RMSD curve indicate docked complex’s stability, and vice versa. The calculated RMSD of 8.5 Å ± 1 Å indicated no significant variation following convergence over the whole simulation time except for some fluctuation in the beginning between 0 and 100 ns in case of V3-TR2 complex (**Figure 9A**). In case of V3-TLR4 complex, the RMSD was calculated to be 11 Å ± 1 Å and this indicated some deviations in the initial state and a minor variation was observed between 2500 to 3500ns but later on, no significant deviations were observed (**Figure 9B**).” The corrected sentence appears below:

“The RMSD of both the complexes was calculated in the bound conformations of the receptors and vaccine. The RMSD profile of the V3-TLR2 complex showed stabilization after an initial equilibration phase, maintaining fluctuations between 5.6 and 6.4 Å throughout the 100 ns simulation (**Figure 9A**), indicating overall structural stability. RMSF analysis revealed moderate flexibility in V3-TLR2, mainly at the N terminal region (**Figure 9B**). The V3-TLR4 complex also remained stable, with RMSD values mostly between 7 and 8 Å and reaching ~ 9 Å in the end (**Figure 9C**), though it exhibited higher fluctuations during initial equilibration phase. While the RMSF analysis of V3-TLR4 showed more pronounced fluctuations at the N- and C-terminal regions (**Figure 9D**)”.

The authors apologize for this error and state that this does not change the scientific conclusions of the article in any way. The original article has been updated.

