# Supplementary figures and images for: Development of a subunit vaccine against the cholangiocarcinoma causing Opisthorchis viverrini: a computational approach
Source: Front Immunol. 2024 Jul 10;15:1281544. doi: 10.3389/fimmu.2024.1281544 (PMC11266093; doi:10.3389/fimmu.2024.1281544)

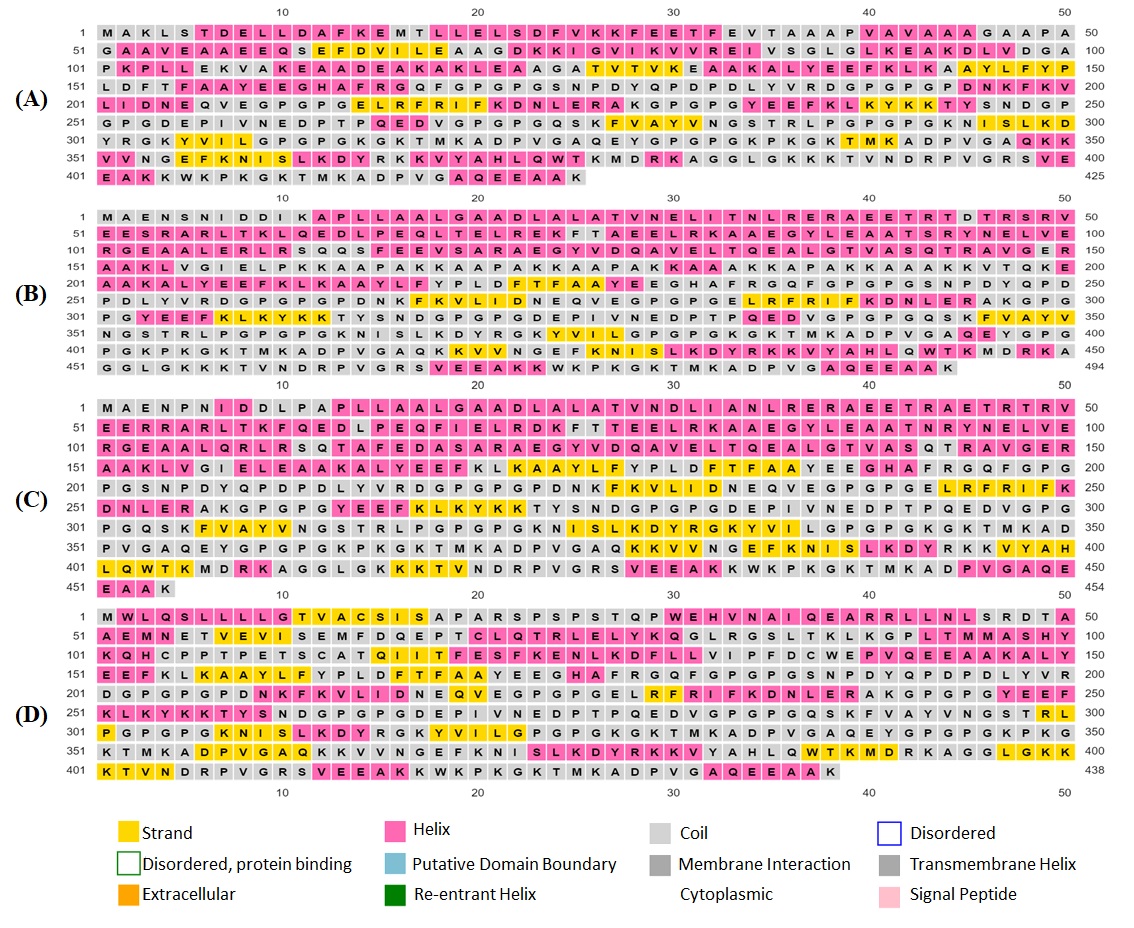

Supplement: Supplementary Figure 1 — Secondary structure of the Vaccines [V1 (A), V2 (B), V3 (C), V4 (D)] containing α-helix, random coil, and β-strand. Pink shows the α-helix, yellow the β-stands and grey the coil regions of the designed vaccines. [file Image_1.jpeg]

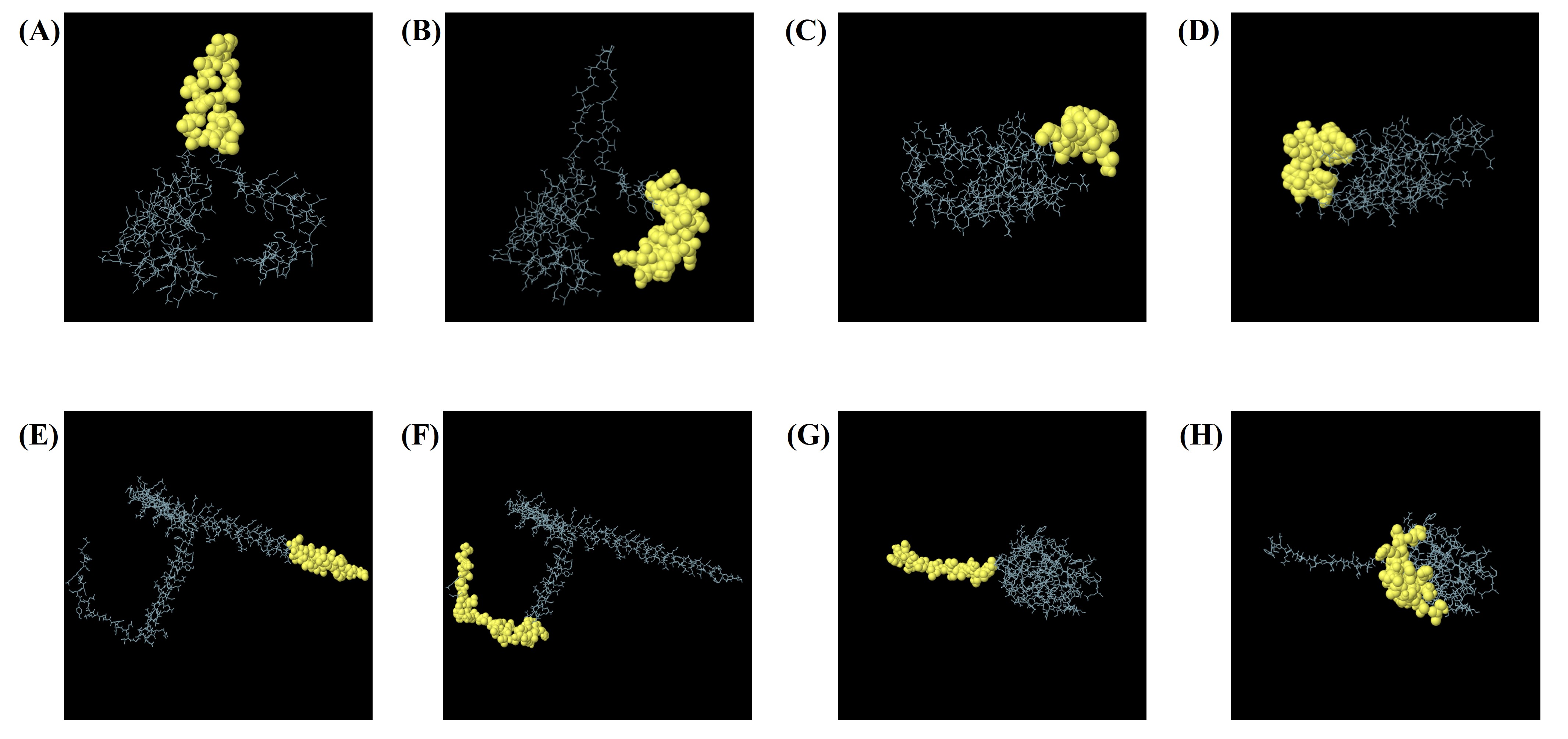

Supplement: Supplementary Figure 2 — Residues of vaccine involved in conformational B cell epitope formation. Vaccine 1 [(A) 0.76 (B) 0.777]; vaccine 2 [(C) 0.844 (D) 0.738]; vaccine 3 [(E) 0.848 (F) 0.796]; Vaccine 4 [(G) 0.837 (H) 0.709] [file Image_2.jpeg]

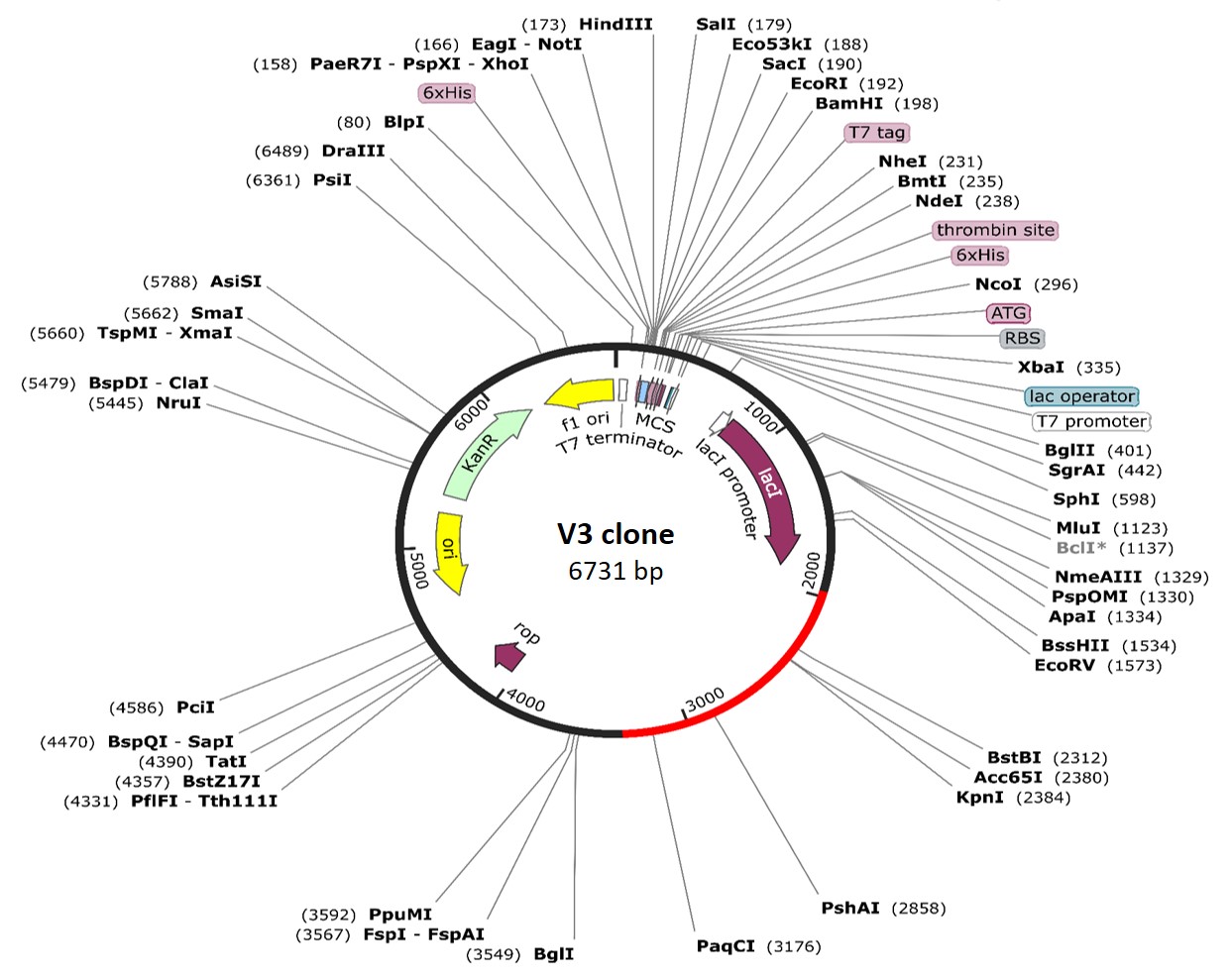

Supplement: Supplementary Figure 3 — In-silico cloning of the final prioritized vaccine V3 in the pet28-a(+) vector. The black color represents the plasmid sequence while the red color indicates the vaccine sequence. [file Image_3.jpeg]
