# Supplementary material for: Development of a subunit vaccine against the cholangiocarcinoma causing Opisthorchis viverrini: a computational approach
Source: Front Immunol. 2024 Jul 10;15:1281544. doi: 10.3389/fimmu.2024.1281544 (PMC11266093; doi:10.3389/fimmu.2024.1281544)
Supplement: Supplementary file 4 [file Table_1.docx]

**Supplementary Table S1.** Analysis and Selection of target CTL epitopes for Thioredoxin Peroxidase (Ov-TPx-1) protein.

| **Allele** | **Start** | **End** | **Peptide** | **Score** | **Rank** | **Antigenicity** | **Allergenicity** | **Toxicity** | **Immunogenicity** |
| --- | --- | --- | --- | --- | --- | --- | --- | --- | --- |
| HLA-B*35:01 | 21 | 29 | LPNQPAPEF | 0.990244 | 0.01 | -0.1752  Non-antigen | Non-allergen | Non-toxin | -0.04349 |
| **HLA-A*23:01** | **55** | **63** | **LFYPLDFTF** | **0.95344** | **0.01** | **1.2351**  **Antigen** | **Non-allergen** | **Non-toxin** | **0.1192** |
| HLA-A*01:01 | 198 | 207 | KADPVGAQEY | 0.942379 | 0.02 | 0.4491  Non-antigen | Allergen | Non-toxin | 0.06681 |
| HLA-A*03:01 | 93 | 102 | SVYAHLQWTK | 0.933078 | 0.02 | 1.0767  Antigen | Allergen | Non-toxin | 0.15945 |
| HLA-A*68:01 | 156 | 164 | TVNDRPVGR | 0.957204 | 0.03 | 0.2644  Non-antigen | Allergen | Non-toxin | 0.11482 |
| HLA-A*03:01 | 112 | 121 | KMNIPLLSDK | 0.877709 | 0.04 | 0.5717  Antigen | Allergen | Non-toxin | -0.02688 |
| HLA-B*15:01 | 43 | 51 | SLKDYRGKY | 0.748897 | 0.1 | 0.4895  Non-antigen | Allergen | Non-toxin | -0.09996 |
| HLA-A*24:02 | 71 | 79 | AFSDAAEEF | 0.63591 | 0.13 | -0.0686  Non-antigen | Non-allergen | Non-toxin | 0.18655 |
| **HLA-B*44:02** | **135** | **144** | **EEGHAFRGQF** | **0.623756** | **0.13** | **1.2582**  **Antigen** | **Non-allergen** | **Non-toxin** | **0.20529** |
| HLA-B*44:02 | 167 | 176 | EEAIRLLEAF | 0.50427 | 0.19 | -0.3383  Non-antigen | Allergen | Non-toxin | 0.28314 |
