# Supplementary material for: Development of a subunit vaccine against the cholangiocarcinoma causing Opisthorchis viverrini: a computational approach
Source: Front Immunol. 2024 Jul 10;15:1281544. doi: 10.3389/fimmu.2024.1281544 (PMC11266093; doi:10.3389/fimmu.2024.1281544)
Supplement: Supplementary file 5 [file Table_2.docx]

**Supplementary Table S2.** Analysis and selection of MHC-I epitopes of Cathepsin F1 (*Ov-CF-1*) protein.

| **Allele** | **Start** | **End** | **Peptide** | **Score** | **Rank** | **Antigenicity** | **Allergenicity** | **Toxicity** | **Immunogenicity** |
| --- | --- | --- | --- | --- | --- | --- | --- | --- | --- |
| HLA-A*01:01 | 70 | 78 | TSEEFKTRY | 0.964294 | 0.01 | 1.0538  Antigen | Allergen | Non-toxin | 0.10725 |
| HLA-A*30:01 | 210 | 218 | STRLPWCEK | 0.837352 | 0.01 | -0.2751  Non-antigen | Allergen | Toxin | 0.21635 |
| **HLA-A*03:01** | **16** | **24** | **ALYEEFKLK** | **0.931126** | **0.02** | **1.2294**  **Antigen** | Non-allergen | **Non-toxin** | **0.11877** |
| HLA-B*51:01 | 167 | 175 | YPPQTYSAI | 0.871592 | 0.02 | 0.2191  Non-antigen | Allergen | Non-toxin | -0.2026 |
| HLA-A*01:01 | 189 | 197 | YTGKDGICY | 0.877814 | 0.04 | -1.7053  Non-antigen | Allergen | Non-toxin | -0.07168 |
| HLA-B*58:01 | 98 | 107 | VTMDNSNFDW | 0.919066 | 0.05 | -0.2000  Non-antigen | Allergen | Non-toxin | -0.09641 |
| HLA-B*35:01 | 52 | 61 | QAMEQGTAEY | 0.861809 | 0.06 | 0.1397  Non-antigen | Non-allergen | Non-toxin | 0.09201 |
| HLA-A*31:01 | 39 | 47 | RIFKDNLER | 0.784597 | 0.07 | 0.5188  Antigen | Allergen | Non-toxin | -0.11435 |
| HLA-A*01:01 | 29 | 38 | YSNDDDELRF | 0.632576 | 0.12 | 0.8040  Antigen | Allergen | Non-toxin | 0.17855 |
| HLA-B*58:01 | 129 | 138 | SVIGNVEGQW | 0.740515 | 0.15 | 0.5040  Antigen | Allergen | Non-toxin | 0.16637 |
