# Supplementary material for: Development of a subunit vaccine against the cholangiocarcinoma causing Opisthorchis viverrini: a computational approach
Source: Front Immunol. 2024 Jul 10;15:1281544. doi: 10.3389/fimmu.2024.1281544 (PMC11266093; doi:10.3389/fimmu.2024.1281544)
Supplement: Supplementary file 6 [file Table_3.docx]

**Supplementary Table S3.** Analysis and selection of target CTL epitopes of calreticulin (*Ov-CALR*) protein.

| **Allele** | **Start** | **End** | **Peptide** | **Score** | **Rank** | **Antigenicity** | **Allergenicity** | **Toxicity** | **Immunogenicity** |
| --- | --- | --- | --- | --- | --- | --- | --- | --- | --- |
| HLA-A*03:01 | 144 | 152 | KVHAIFSYK | 0.974019 | 0.01 | -0.3391 (Non-antigen) | Non-allergen | Non-toxin | 0.14789 |
| HLA-B*58:01 | 312 | 320 | IAHVGFELW | 0.973599 | 0.01 | 0.8721 (Antigen) | Allergen | Non-toxin | 0.27326 |
| HLA-A*03:01 | 112 | 121 | KLLPSSIDQK | 0.972732 | 0.01 | 0.3591 (Non-antigen) | Allergen | Non-toxin | -0.26064 |
| HLA-A*33:01 | 271 | 279 | SAYAEVYFY | 0.854339 | 0.02 | 1.0785 (Antigen) | Allergen | Non-toxin | 0.23981 |
| HLA-B*58:01 | 42 | 50 | RYYGISRKL | 0.963427 | 0.03 | -1.3297 (Non-antigen) | Allergen | Non-toxin | -0.07555 |
| HLA-B*35:01 | 13 | 21 | LPSSIDQKTF | 0.920319 | 0.03 | 0.5656 (Antigen) | Allergen | Non-toxin | -0.33733 |
| **HLA-A*11:01** | **333** | **341** | **KTSADARYY** | **0.879874** | **0.03** | **1.0587 (Antigen)** | Non-allergen | **Non-toxin** | **0.08562** |
| HLA-A*23:01 | 73 | 81 | RVWRPRNKL | 0.877331 | 0.03 | 0.6354 (Antigen) | Allergen | Non-toxin | 0.03044 |
| HLA-A*32:01 | 346 | 354 | AEVYFYEHF | 0.719534 | 0.03 | 1.4834 (Antigen) | Allergen | Non-toxin | 0.2236 |
| HLA-B*07:02 | 178 | 186 | YLKLLPSSI | 0.922428 | 0.04 | 1.0032 (Antigen) | Allergen | Non-toxin | -0.33868 |
