# Supplementary material for: Development of a subunit vaccine against the cholangiocarcinoma causing Opisthorchis viverrini: a computational approach
Source: Front Immunol. 2024 Jul 10;15:1281544. doi: 10.3389/fimmu.2024.1281544 (PMC11266093; doi:10.3389/fimmu.2024.1281544)
Supplement: Supplementary file 7 [file Table_4.docx]

**Supplementary Table S4.** Analysis and Selection of target HTL epitopes for Thioredoxin Peroxidase (Ov-TPx-1) protein.

| **Allele** | **Start** | **End** | **Peptide** | **Score** | **Rank** | **Antigenicity** | **Allergenicity** | **IFN inducer** | **IL4 pred** | **IL10 pred** |
| --- | --- | --- | --- | --- | --- | --- | --- | --- | --- | --- |
| HLA-DQA1*01:01/DQB1*05:01 | 66 | 80 | PTELIAFSDAAEEFK | 0.1205 | 0.01 | 0.4090  Non-antigen | Allergen | Negative | Inducer | Inducer |
| HLA-DRB1*12:01 | 166 | 180 | VEEAIRLLEAFHFHD | 0.9538 | 0.01 | -0.1695  Non-antigen | Allergen | Negative | Inducer | Inducer |
| HLA-DRB1*11:01 | 97 | 111 | HLQWTKMDRKAGGLG | 0.9668 | 0.06 | 0.6555  Antigen | Allergen | Negative | Inducer | Inducer |
| HLA-DRB1*09:01 | 120 | 134 | DKNMKISRAYHVLDE | 0.8529 | 0.07 | 0.0006  Non-antigen | Non-allergen | Negative | Inducer | Inducer |
| **HLA-DRB1*15:01** | **40** | **54** | **KNISLKDYRGKYVIL** | **0.8455** | **0.35** | **0.7776**  **Antigen** | **Non-allergen** | **Negative** | **Inducer** | **Inducer** |
| HLA-DRB3*01:01 | 140 | 154 | FRGQFLIDPKGILRQ | 0.6735 | 0.46 | 0.5488  Antigen | Allergen | Negative | Inducer | Inducer |
| HLA-DRB1*04:01 | 14 | 28 | LVNAMALLPNQPAPE | 0.7957 | 0.77 | 0.4055  Non-antigen | Allergen | Negative | Non-inducer | Inducer |
| **HLA-DRB1*03:01** | **193** | **207** | **KGKTMKADPVGAQEY** | **0.7742** | **0.96** | **0.7638**  **Antigen** | **Non-allergen** | **Negative** | **Inducer** | **Inducer** |
| **HLA-DQA1*05:01/DQB1*02:01** | **191** | **205** | **KPKGKTMKADPVGAQ** | **0.4955** | **1.2** | **1.2851**  **Antigen** | **Non-allergen** | Negative | Inducer | Inducer |
