# Supplementary material for: Development of a subunit vaccine against the cholangiocarcinoma causing Opisthorchis viverrini: a computational approach
Source: Front Immunol. 2024 Jul 10;15:1281544. doi: 10.3389/fimmu.2024.1281544 (PMC11266093; doi:10.3389/fimmu.2024.1281544)
Supplement: Supplementary file 8 [file Table_5.docx]

**Supplementary Table S5.** Selected HTL epitopes for Cathepsin F1 (*Ov-CF-1*) protein.

| **Allele** | **Start** | **End** | **Peptide** | **Score** | **Rank** | **Antigenicity** | **Allergenicity** | **IFN inducers** | **IL4 pred** | **IL10 pred** |
| --- | --- | --- | --- | --- | --- | --- | --- | --- | --- | --- |
| **HLA-DQA1*01:01/DQB1*05:01** | **35** | **49** | **ELRFRIFKDNLERAK** | **0.0992** | **0.04** | **1.4244**  **Antigen** | **Non-allergen** | **Negative** | **Inducer** | **Inducer** |
| **HLA-DRB1*13:02** | **18** | **32** | **YEEFKLKYKKTYSND** | **0.7787** | **0.13** | **0.5567**  **Antigen** | **Non-allergen** | **Negative** | **Inducer** | **Inducer** |
| HLA-DPA1*01:03/DPB1*04:01 | 58 | 72 | TAEYGVTQFSDLTSE | 0.6967 | 0.15 | 0.1694  Non-antigen | Allergen | Negative | Non-inducer | Inducer |
| **HLA-DRB1*04:01** | **84** | **98** | **DEPIVNEDPTPQEDV** | **0.8048** | **0.68** | **0.8441**  **Antigen** | **Non-allergen** | **Negative** | **Non-inducer** | **Inducer** |
| HLA-DPA1*01:03/DPB1*02:01 | 130 | 144 | VIGNVEGQWFRKTGD | 0.583 | 0.79 | 0.3519  Non-antigen | Non-allergen | Negative | Non-inducer | Inducer |
| HLA-DRB1*01:01 | 172 | 186 | YSAIEEMGGLELRSD | 0.8275 | 0.8 | 0.0079  Non-antigen | Allergen | Negative | Inducer | Inducer |
| **HLA-DRB1*15:01** | **200** | **214** | **QSKFVAYVNGSTRLP** | **0.7214** | **0.85** | **0.5978**  **Antigen** | **Non-allergen** | **Negative** | **Inducer** | **Inducer** |
| HLA-DQA1*01:01/DQB1*05:01 | 101 | 115 | DNSNFDWRDHGAVGP | 0.0254 | 1.6 | 1.2799  Antigen | Allergen | Negative | Inducer | Inducer |
