# Supplementary material for: Development of a subunit vaccine against the cholangiocarcinoma causing Opisthorchis viverrini: a computational approach
Source: Front Immunol. 2024 Jul 10;15:1281544. doi: 10.3389/fimmu.2024.1281544 (PMC11266093; doi:10.3389/fimmu.2024.1281544)
Supplement: Supplementary file 9 [file Table_6.docx]

**Supplementary Table S6.** Analysis and Selection of target HTL epitopes of calreticulin (*Ov-CALR )* protein.

| Allele | Start | End | Peptide | Score | Rank | Antigenicity | Allergenicity | IFN-inducers | IL4 pred | IL10 pred |
| --- | --- | --- | --- | --- | --- | --- | --- | --- | --- | --- |
| HLA-DRB3*01:01 | **296** | **310** | **SNPDYQPDPDLYVRD** | **0.966** | **0.01** | **1.0343**  **antigen** | **Non-allergen** | **Negative** | **Non-inducer** | **Inducer** |
| HLA-DRB1*04:01 | **179** | **193** | **PDNKFKVLIDNEQVE** | **0.9738** | **0.01** | **0.9647**  **antigen** | **Non-allergen** | **Negative** | **Non-inducer** | **Inducer** |
| HLA-DPA1*02:01/DPB1*01:01 | 15 | 29 | YAEVYFYEHFSKDSI | 0.467 | 0.02 | 0.7114  antigen | Allergen | Negative | Non-inducer | Inducer |
| HLA-DRB1*01:01 | 336 | 350 | DPQFAKEEGERVWRP | 0.9714 | 0.08 | -0.1896  non-antigen | Allergen | Negative | Non-inducer | Inducer |
| HLA-DRB1*13:02 | 146 | 160 | HAIFSYKGKNHLIKK | 0.7843 | 0.12 | -0.4440  non-antigen | Non-allergen | Negative | Inducer | Inducer |
| HLA-DRB1*01:01 | 107 | 121 | GGAYLKLLPSSIDQK | 0.9516 | 0.2 | 0.1850  non-antigen | Allergen | Negative | Non-inducer | Inducer |
| HLA-DRB1*08:02 | 70 | 84 | ADARYYGISRKLDKP | 0.8391 | 0.21 | -0.1628  non-antigen | Allergen | Negative | Inducer | Inducer |
| HLA-DRB1*12:01 | 89 | 103 | DKTLVVQYIVKYEQS | 0.7145 | 0.5 | 0.3266  non-antigen | Non-allergen | Negative | Inducer | Inducer |
| HLA-DRB1*01:01 | 197 | 211 | LEDDWDMLLPKEIDD | 0.8605 | 0.61 | 0.3831  non-antigen | Allergen | Negative | Non-inducer | Inducer |
| HLA-DRB1*07:01 | 316 | 330 | GFELWQVTSGSVFDE | 0.7574 | 0.73 | -0.2854  non-antigen | Non-allergen | Negative | Inducer | Inducer |
