# Supplementary material for: Development of a subunit vaccine against the cholangiocarcinoma causing Opisthorchis viverrini: a computational approach
Source: Front Immunol. 2024 Jul 10;15:1281544. doi: 10.3389/fimmu.2024.1281544 (PMC11266093; doi:10.3389/fimmu.2024.1281544)
Supplement: Supplementary file 10 [file Table_7.docx]

**Supplementary Table S7.** Predicted B-cell epitopes from Bepipred Linear Epitope prediction 2.0 method.

| **Protein** | **Start** | **End** | **Peptide** | **Length** | **Antigenicity** | **Allergenicity** | **Toxicity** |
| --- | --- | --- | --- | --- | --- | --- | --- |
| Calreticulin | 25 | 46 | SKDSIGDKPGQWVESNHHSDET | 22 | 0.7223 (Antigen) | Allergen | Non-toxin |
|  | 152 | 166 | KGKNHLIKKEVRCKD | 15 | 0.5690 (Antigen) | Allergen | Non-toxin |
| Cathepsin F1 | 23 | 36 | LKYKKTYSNDDDEL | 14 | 0.4006 (Non-antigen) | Non-allergen | Non-toxin |
|  | 49 | 59 | KRLQAMEQGTA | 11 | 0.3375 (Non-antigen) | Non-allergen | Non-toxin |
|  | 140 | 149 | RKTGDLLGLS | 10 | -0.1413 (Non-antigen) | Non-allergen | Non-toxin |
|  | 179 | 200 | GGLELRSDYPYTGKDGICYMDQ | 22 | -0.2421 (Non-antigen) | Non-allergen | Non-toxin |
| Thioredoxin Peroxidase | 14 | 32 | LVNAMALLPNQPAPEFSGM | 19 | 0.2846 (Non-antigen) | Non-allergen | Non-toxin |
|  | **34** | **48** | **VVNGEFKNISLKDYR** | **15** | **1.3446 (Antigen)** | **Non-allergen** | **Non-toxin** |
|  | **94** | **112** | **VYAHLQWTKMDRKAGGLGK** | **19** | **0.6168 (Antigen)** | **Non-allergen** | **Non-toxin** |
|  | **156** | **169** | **TVNDRPVGRSVEEA** | **14** | **0.6529 (Antigen)** | **Non-allergen** | **Non-toxin** |
|  | **190** | **206** | **WKPKGKTMKADPVGAQE** | **17** | **1.0614(Antigen)** | **Non-allergen** | **Non-toxin** |
