# Supplementary material for: Development of a subunit vaccine against the cholangiocarcinoma causing Opisthorchis viverrini: a computational approach
Source: Front Immunol. 2024 Jul 10;15:1281544. doi: 10.3389/fimmu.2024.1281544 (PMC11266093; doi:10.3389/fimmu.2024.1281544)
Supplement: Supplementary file 11 [file Table_8.docx]

| **Vaccine construct** | **Residues** | **No. of residues** | **Score** |
| --- | --- | --- | --- |
| V1 | A:M1, A:A2, A:K3, A:L4, A:S5, A:T6, A:D7, A:E8, A:L9, A:L10, A:D11, A:A12, A:F13, A:K14, A:E15, A:M16, A:T17, A:L18, A:L19, A:E20, A:L21, A:S22, A:D23, A:F24, A:K26, A:K27  A:V40, A:A41, A:V42, A:A43, A:A44, A:A45, A:G46, A:A47, A:A48, A:P49, A:A50, A:G51, A:A52, A:A53, A:V54, A:E55, A:A56, A:A57, A:E58 | 26  19 | 0.76  0.777 |
| V2 | A:Q128, A:A129, A:V130, A:E131, A:L132, A:T133, A:Q134, A:E135, A:A136, A:L137, A:G138  A:F75, A:T76, A:A77, A:E78, A:E79, A:L80, A:R81, A:A83, A:A84, A:E85, A:G86, A:Y87, A:L88, A:E89, A:A90, A:A91, A:T92, A:S93, A:R94, A:N96, A:E97, A:R101 | 11  22 | 0.844  0.738 |
| V3 | A:M1, A:A2, A:E3, A:N4, A:P5, A:N6, A:I7, A:D8, A:D9, A:L10, A:P11, A:A12, A:P13, A:L14, A:L15, A:A16, A:A17, A:L18, A:G19, A:A20, A:A21, A:D22, A:L23  A:A146, A:G148, A:E149, A:R150, A:A151, A:A152, A:K153, A:L154, A:V155, A:G156, A:I157, A:E158, A:L159, A:E160, A:A161, A:A162, A:K163, A:A164, A:L165, A:Y166, A:E168, A:F169, A:L171, A:K172, A:A173, A:A174 | 23  26 | 0.848  0.796 |
| V4 | A:M1, A:W2, A:L3, A:Q4, A:S5, A:L6, A:L7, A:L8, A:L9, A:G10, A:T11, A:V12, A:A13, A:C14, A:S15, A:I16, A:S17, A:A18, A:P19, A:A20  A:N44, A:L45, A:S46, A:R47, A:D48, A:T49, A:A50, A:A51, A:E52, A:M53, A:N54, A:E55, A:T56, A:G81, A:L82, A:R83, A:G84, A:S85, A:I116, A:T118, A:F119, A:E120 | 20  22 | 0.837  0.709 |

**Supplementary Table S8.** Vaccine’s residues involved in the formation of Conformational B-cell epitopes.
